# Association of FKBP51 with Priming of Autophagy Pathways and Mediation of Antidepressant Treatment Response: Evidence in Cells, Mice, and Humans

**DOI:** 10.1371/journal.pmed.1001755

**Published:** 2014-11-11

**Authors:** Nils C. Gassen, Jakob Hartmann, Jürgen Zschocke, Jens Stepan, Kathrin Hafner, Andreas Zellner, Thomas Kirmeier, Lorenz Kollmannsberger, Klaus V. Wagner, Nina Dedic, Georgia Balsevich, Jan M. Deussing, Stefan Kloiber, Susanne Lucae, Florian Holsboer, Matthias Eder, Manfred Uhr, Marcus Ising, Mathias V. Schmidt, Theo Rein

**Affiliations:** 1 Department of Translational Research in Psychiatry, Max Planck Institute of Psychiatry, Munich, Germany; 2 Department of Stress Neurobiology and Neurogenetics, Max Planck Institute of Psychiatry, Munich, Germany; 3 Department of Clinical Research, Max Planck Institute of Psychiatry, Munich, Germany; Mount Sinai School of Medicine, United States of America

## Abstract

**Background:**

FK506 binding protein 51 (FKBP51) is an Hsp90 co-chaperone and regulator of the glucocorticoid receptor, and consequently of stress physiology. Clinical studies suggest a genetic link between FKBP51 and antidepressant response in mood disorders; however, the underlying mechanisms remain elusive. The objective of this study was to elucidate the role of FKBP51 in the actions of antidepressants, with a particular focus on pathways of autophagy.

**Methods and Findings:**

Established cell lines, primary neural cells, human blood cells of healthy individuals and patients with depression, and mice were treated with antidepressants. Mice were tested for several neuroendocrine and behavioral parameters. Protein interactions and autophagic pathway activity were mainly evaluated by co-immunoprecipitation and Western blots. We first show that the effects of acute antidepressant treatment on behavior are abolished in FKBP51 knockout (51KO) mice. Autophagic markers, such as the autophagy initiator Beclin1, were increased following acute antidepressant treatment in brains from wild-type, but not 51KO, animals. FKBP51 binds to Beclin1, changes decisive protein interactions and phosphorylation of Beclin1, and triggers autophagic pathways. Antidepressants and FKBP51 exhibited synergistic effects on these pathways. Using chronic social defeat as a depression-relevant stress model in combination with chronic paroxetine (PAR) treatment revealed that the stress response, as well as the effects of antidepressants on behavior and autophagic markers, depends on FKBP51. In human blood cells of healthy individuals, FKBP51 levels correlated with the potential of antidepressants to induce autophagic pathways.

Importantly, the clinical antidepressant response of patients with depression (*n* = 51) could be predicted by the antidepressant response of autophagic markers in patient-derived peripheral blood lymphocytes cultivated and treated ex vivo (Beclin1/amitriptyline: *r* = 0.572, *p* = 0.003; Beclin1/PAR: *r* = 0.569, *p* = 0.004; Beclin1/fluoxetine: *r* = 0.454, *p* = 0.026; pAkt/amitriptyline: *r* = −0.416, *p* = 0.006; pAkt/PAR: *r* = −0.355, *p* = 0.021; LC3B-II/PAR: *r* = 0.453, *p* = 0.02), as well as by the lymphocytic expression levels of FKBP51 (*r* = 0.631, *p*<0.0001), pAkt (*r* = −0.515, *p* = 0.003), and Beclin1 (*r* = 0.521, *p* = 0.002) at admission. Limitations of the study include the use of male mice only and the relatively low number of patients for protein analyses.

**Conclusions:**

To our knowledge, these findings provide the first evidence for the molecular mechanism of FKBP51 in priming autophagic pathways; this process is linked to the potency of at least some antidepressants. These newly discovered functions of FKBP51 also provide novel predictive markers for treatment outcome, consistent with physiological and potential clinical relevance.

*Please see later in the article for the Editors' Summary*

## Abstract 2

Theo Rein and colleagues examine the role of FKBP51 in the actions of antidepressants, with a particular focus on pathways of autophagy.

*Please see later in the article for the Editors' Summary*

## Editors' Summary

**Background:**

Everyone feels miserable sometimes, but about one in six people will have an episode of clinical depression during their lifetime. For people who are clinically depressed, overwhelming feelings of sadness, anxiety, and hopelessness can last for months or years. Affected individuals lose interest in activities they used to enjoy, they sometimes have physical symptoms such as disturbed sleep, and they may contemplate suicide. Clinicians diagnose depression and determine its severity using questionnaires (“depression rating scales”) that explore the patient's feelings and symptoms. Mild depression is often treated with talking therapies (psychotherapy) such as cognitive behavioral therapy, which helps people change negative ways of thinking. For more severe depression, patients are also usually prescribed an antidepressant, most commonly a “selective serotonin reuptake inhibitor” such as paroxetine or a tricyclic antidepressant such as amitriptyline.

**Why Was This Study Done?:**

Unfortunately, antidepressants don't work for more than half of patients. Moreover, because it is unclear how antidepressants work, it is not possible to predict which patients will respond to which antidepressants. Thus, matching patient to drug can be a lengthy, sometimes unsuccessful, process. Here, the researchers use several approaches to test the hypothesis that a protein called FK506 binding protein 51 (FKBP51) is involved in the actions of antidepressants and to investigate whether the ability of both FKBP51 and antidepressants to regulate a process called autophagy underlies the impact of FKBP51 on antidepressant responses. FKBP51 is a regulator of stress physiology, which is connected to the development and treatment of depression; genetic studies have suggested a link between FKBP51 expression and the antidepressant response rate. Some antidepressants are known to alter the initial steps in the autophagy pathway, a multistep process that maintains the integrity of cells through regulated degradation and recycling of cellular components; however, the potential synergistic role of FKBP51 and antidepressants in regulating pathways of autophagy are unknown.

**What Did the Researchers Do and Find?:**

The researchers first treated wild-type mice and FKBP51 knockout mice (genetically altered animals that make no FKBP51) with an acute dose of antidepressant and compared their behavior in a forced swim test, an assay that measures the action of antidepressants in mice by determining how long the mice struggle or float inertly when placed in deep water. As expected, acute antidepressant treatment increased the time that wild-type mice spent struggling. However, this effect of antidepressant treatment was greatly attenuated in the FKBP51 knockout mice. Moreover, the levels of several autophagy markers increased in the brains of wild-type mice following antidepressant treatment but not in the brains of FKBP51 knockout mice. Next, using “chronic social defeat stress” to model the “endophenotype” of depression (a combination of physiological, hormonal, and behavioral traits seen in people with depression) in mice, the researchers showed that the stress response and the effect of chronic antidepressants on behavior and on autophagic markers all depend on FKBP51. Using cell-based assays, the researchers showed that antidepressants and FKBP51 had synergistic (interactive) effects on the autophagic pathway and that, in human blood cells, FKBP51 levels correlated with the potential of antidepressants to induce autophagic pathways. Finally, the researchers report that the clinical response to antidepressant treatment in 51 patients with depression was associated with the response of autophagic markers in their peripheral blood lymphocytes to antidepressant treatment in test tubes, and that the expression levels of FKBP51 and autophagy markers in patient lymphocytes at admission were associated with subsequent clinical responses to antidepressants.

**What Do These Findings Mean?:**

These findings suggest that the protein FKBP51 is required for the effects of both acute and chronic treatment with some antidepressants on behavior and on autophagic pathways in mice. These findings also reveal an association between antidepressant treatment responses in patients and both the expression levels of FKBP51 and autophagy markers in lymphocytes at admission and the response of autophagic markers to antidepressant treatment in patient lymphocytes. The accuracy of these findings is limited by the small number of clinical samples available for analysis, by the use of only male mice in the animal experiments, and by the inability of animal models of depression to fully replicate the human condition. Nevertheless, these findings identify the early stages of autophagy as potential targets for the development of new antidepressants and identify several potential biomarkers that might, after further clinical validation, help clinicians predict antidepressant efficacy in patients with depression.

**Additional Information:**

Please access these websites via the online version of this summary at http://dx.doi.org/10.1371/journal.pmed.1001755.

- The US National Institute of Mental Health provides information on all aspects of depression (in English and Spanish), including information on antidepressants
- The UK National Health Service Choices website provides detailed information about depression and about antidepressants; it also provides personal stories about depression
- The UK charity Mind provides information on depression, including some personal stories about depression
- More personal stories about depression are available from healthtalk.org
- MedlinePlus provides links to other resources about depression
- Wikipedia has a page on autophagy (note that Wikipedia is a free online encyclopedia that anyone can edit; available in several languages)
- The patients included in this study were all enrolled in the Munich Antidepressant Response Signature project, which aims to identify gene variants and biomarkers that predict treatment outcomes with antidepressants

## Introduction

Depression is the most common mental disorder, affecting an estimated 350 million people worldwide [1]. While currently available antidepressants show an overall favorable benefit/risk balance, less than 50% of patients achieve sustained remission following treatment, indicating a large inter-individual variability in treatment efficacy and response [2]. The poor effectiveness of antidepressants is at least partially due to the fact that the mechanism of action of these drugs, which is responsible for their clinical potency, is still largely unknown. As a consequence, it is currently also not possible to predict treatment success in individual patients, confining physicians and patients to lengthy trial and error procedures with uncertain success.

FK506 binding protein 51 (FKBP51) is a regulator of the glucocorticoid receptor, and consequently of the stress hormone axis and stress physiology [3]–[7]. Human genetic studies have suggested a link between FKBP51 and antidepressant response rate [8]–[11]. Despite the intimate connection of stress physiology to the pathophysiology and treatment of depression [12],[13], the mechanistic role of FKBP51 in antidepressant response has not been elucidated.

Putative convergent molecular pathways common to both FKBP51 and antidepressants might explain and substantiate the suggested impact of FKBP51 on antidepressant response; the modulation of these pathways by FKBP51 could influence their reactivity to antidepressants. We analyzed whether these pathways involve autophagic events based on reports stating that some antidepressants alter at least the initial processes of autophagy [14],[15]. Given that FKBP51 can act via the central chaperone Hsp90, which controls several kinases including the autophagy regulators Akt and Beclin1 [16], intracellular autophagic events appeared more likely than membrane targets of antidepressants to be co-regulated by FKBP51.

Autophagy is a conserved cellular degradation process ensuring continuous removal of damaged macromolecules and, thus, the functional integrity of cells and tissues [17]. The predominant form of autophagy involves the stepwise formation of an autophagosome, which engulfs damaged cytosolic proteins or organelles, and subsequent fusion of the autophagosome with a lysosome to yield an autolysosome. This process is orchestrated by a series of autophagy-related genes (Atg genes) and is regulated by complex pathways that are partly under the control of the kinase mTOR (mammalian target of rapamycin) [18]. Based on the multitude of involved factors, autophagy is commonly assessed by the determination of several autophagy executers. These include, among others, the initiators Beclin1 and Vps34 (class III phosphatidylinositol-3-kinase); Atg12 and LC3B-II (derived from LC3B-I upon lipidation), which are involved in autophagosome membrane expansion and vesicle formation; and LC3B-II in association with mature autophagosomes. Very recently, the mTOR-independent regulation of autophagy was reported: Akt-dependent phosphorylation of Beclin1 shifts Beclin1's action from autophagy to apoptosis [19]. As the guardian of cellular homeostasis, autophagy has been established as a pivotal process in a range of (patho-)physiological conditions, including infectious diseases, cancer, diabetes, and, more recently, neurotransmission and neurodegeneration [20]–[23]. Indirect evidence reveals a potential role of autophagy in depression; some antidepressants change the autophagic flux and the expression of autophagy markers [14],[15],[24]–[26]. Moreover, the induction of autophagy by the mTOR inhibitor rapamycin was reported to exert antidepressant-like effects in mice [27].

Assessing depression and the action of antidepressants in animals is very challenging. Animals lack not only measurable consciousness of self, self-reflection, and consideration of others, but also hallmarks of the disorder such as depressed mood, guilt, suicidality, or low self-esteem. Nonetheless, depression encompasses so-called endophenotypes that can be reproduced independently and evaluated in animals, including physiological, endocrine, and behavioral traits [28],[29]. Chronic social defeat stress (CSDS) [5],[30],[31] is a repeatedly validated paradigm used to model specific endophenotypes of depression [28]. To mimic the clinical treatment situation, chronic stress paradigms can be combined with chronic antidepressant treatment during the recovery period after chronic stress exposure. In addition, the forced swim test (FST) is a simple and widely used assay to measure the actions of antidepressants in mice.

The objective of this study was to elucidate the role of FKBP51 in the actions of antidepressants using a broad interdisciplinary approach combining methods from molecular and cell biology, animal behavior, and clinical science. More specifically, we aimed to test the hypothesis that the actions of FKBP51 and antidepressants might synergize in regulating pathways of autophagy and that this synergy might link to clinical treatment success.

## Methods

### Cell lines

Human embryonic kidney cells (HEK-293; ATCC, CRL-1573) and mouse embryonic fibroblasts (MEFs) were maintained in DMEM (Gibco) supplemented with 10% FCS and 100 units/ml penicillin and streptomycin. FKBP51 knockout (51KO) and Akt1/2 knockout (Akt1/2KO) MEFs (kind gift of Nissim Hay, University of Illinois) have been described before [6],[32].

### Primary Cultures of Rat Primary Neurons and Astrocytes

Neocortical neurons were enriched from embryonic day 18 rat brains, and enriched astroglial cultures were prepared from postnatal day 1 rat pups (Sprague-Dawley, Charles River) and handled as previously described [33],[34].

### FKBP51 Rescue in MEF 51KO cells

MEF cells were detached from the plate, and 2×10^6^ cells were immediately resuspended in 100 µl of transfection buffer (50 mM HEPES [pH 7.3], 90 mM NaCl, 5 mM KCl, 0.15 mM CaCl_2_) [35]. A maximal amount of 5 µg of plasmid DNA was added to the cell suspension, and electroporation was carried out using the Amaxa Nucleofector system (program # T-020). Cells were replated at a density of 10^5^ cells/cm^2^ and further processed for Western blot analysis.

### Chemicals for Treatments

The following drugs were used: amitriptyline hydrochloride (Sigma, A8404), bafilomycin A_1_ (Alfa Aeser, J61835), dexamethasone (Sigma, D1756), fluoxetine hydrochloride (Sigma, F132), haloperidol (Sigma, H1512), ketamine hydrochloride (Sigma, K2753), paroxetine hydrochloride hemihydrate (Sigma, P9623), and scopolamine hydrobromide (Sigma, S1875).

### Plasmids

Plasmids expressing FKBP51-FLAG, Akt1, Akt2, and LC3B have been described previously [7],[36]–[38]. The Akt1 and Akt2 plasmids were a kind gift from H. Lee, Moffitt Cancer Center.

### Co-immunoprecipitation

Co-immunoprecipitations (CoIPs) of FLAG-tagged FKBP51/52 and FLAG-tagged FKBP51 with endogenous Akt were performed in HEK-293 cells. 5×10^6^ cells were electroporated with 5 µg of the respective expression plasmids using a GenePulser (Bio-Rad) at 350 V/700 µF in 400 µl of electroporation buffer (50 mM K_2_HPO_4_/KH_2_PO_4_, 20 mM KAc [pH 7.35], 25 mM MgSO_4_). After 3 d of cultivation in DMEM/10% FCS, cells were lysed in CoIP buffer containing 20 mM Tris-HCl (pH 8.0), 100 mM NaCl, 1 mM EDTA, and 0.5% Igepal, complemented with protease inhibitor cocktail (Sigma, P2714). This was followed by incubation on an overhead shaker for 20 min at 4°C. The lysate was cleared by centrifugation, the protein concentration was determined, and 1.2 mg of lysate was incubated with 2.5 µg of FLAG antibody overnight at 4°C. 20 µl of BSA-blocked Protein G Dynabeads (Invitrogen, 100-03D) were added to the lysate-antibody mix, followed by 3 h of incubation at 4°C. The beads were washed three times with PBS, and protein-antibody complexes were eluted with 100 µl of 1× FLAG-peptide solution (Sigma, 100–200 µg/ml, F3290) in CoIP buffer for 30 min at 4°C. 5–15 µg of the cell lysates or 2.5 µl of the immunoprecipitates was separated by SDS-PAGE.

### Western Blot Analysis

Protein extracts were obtained by lysing cells in 62.5 mM Tris, 2% SDS, and 10% sucrose, supplemented with protease (Sigma, P2714) and phosphatase (Roche, 04906837001) inhibitor cocktail. Samples were sonicated and heated at 95°C for 5 min. Proteins were separated by SDS-PAGE and electro-transferred onto nitrocellulose membranes. Blots were placed in Tris-buffered saline, supplemented with 0.05% Tween (Sigma, P2287) and 5% non-fat milk for 1 h at room temperature and then incubated with primary antibody (diluted in TBS/0.05% Tween) overnight at 4°C. The following primary antibodies were used: Beclin1 (1∶1,000, Cell Signaling, #3495), pBeclin1 (S234 and S295, both 1∶1,000, Phosphosolutions, #p117-234 and #p117-295), Atg12 (1∶1,000, Cell Signaling, #2010), LC3B-II/I (1∶1,000, Cell Signaling, #2775), FLAG (1∶7,000, Rockland, 600-401-383), PI3K Class III (Vps34, 1∶1,000, Cell Signaling, #4263), FKBP51 (1∶1,000, Bethyl, A301-430A), Akt (1∶1,000, Cell Signaling, #4691), pAkt (Ser473 and T308, both 1∶1,000, Cell Signaling, #4058 and #9275), Actin (1∶5,000, Santa Cruz Biotechnology, sc-1616).

Subsequently, blots were washed and probed with the respective horseradish-peroxidase- or fluorophore-conjugated secondary antibody for 1 h at room temperature. The immuno-reactive bands were visualized either using ECL detection reagent (Millipore, WBKL0500) or directly by excitation of the respective fluorophore. Recording of the band intensities was performed with the ChemiDoc MP system from Bio-Rad and X-ray films.

### Quantification of Protein Data

All protein data were normalized to Actin, which was detected on the same blot in the same lane (multiplexing). In the case of Akt phosphorylation, we built the ratio of pAkt^S473^ to total Akt, as indicated. We obtained very similar results leading to the same conclusions when we normalized pAkt^S473^ to Actin. In the case of LC3B-II/I, we also provide the direct ratio of the two isoforms; we also provide normalization of LC3B-II to Actin for a few examples, yielding the same results.

### Animals

All experiments were carried out in the animal facilities of the Max Planck Institute of Psychiatry in Munich, Germany. The 51KO mouse line was previously generated [6] and fully backcrossed (12 generations) to C57BL/6. Genotypes were verified by PCR of tail DNA. Only male mice were used for the experiment, obtained from heterozygous breeding pairs, to avoid an influence of the menstrual cycle on the molecular and behavioral outcomes. Animals were between 10 and 16 wk old at the start of the experiment. Mice were held under standard conditions (12L∶12D light cycle, lights on at 08:00 am, temperature 23±2°C), were singly housed, and were acclimated to the room for 1 wk before the beginning of the experiments. Male CD1 mice (16–18 wk of age) served as resident mice, which were held under the conditions described above. They were allowed to habituate to the social defeat cage for 2 wk before the experiment. Food (Altromin, 1314) and tap water were available ad libitum.

### Acute Animal Treatments and Behavioral Experiments

#### Acute paroxetine treatments

Male mice (age 10–16 wk) were injected intraperitoneally with a single dose of either paroxetine (PAR) (10 mg/kg) or saline vehicle. 45 min later, a subgroup of mice (wild-type/vehicle, *n* = 10; 51KO/vehicle, *n* = 9; wild-type/PAR, *n* = 10; 51KO/PAR, *n* = 10) was sacrificed. Hippocampus and prefrontal cortex were extracted for protein analysis. Another subgroup (wild-type/vehicle, *n* = 8; 51KO/vehicle, *n* = 8; wild-type/PAR, *n* = 9; 51KO/PAR, *n* = 7) was subjected to an FST (test duration 6 min) 45 min after the injection. Another subgroup (wild-type, *n* = 3; 51KO, *n* = 4) was sacrificed, and brain tissue was sampled for analysis of drug concentrations.

#### Acute amitriptyline treatment

Male mice (age 10–16 wk) were injected intraperitoneally with a single dose of either amitriptyline (AMI) (10 mg/kg) or saline vehicle. 45 min later, a subgroup of mice (wild-type/vehicle, *n* = 10; 51KO/vehicle, *n* = 9; wild-type/AMI, *n* = 9; 51KO/AMI, *n* = 11) was subjected to an FST. Another subgroup (wild-type, *n* = 5; 51KO, *n* = 5) was sacrificed, and brain tissue and blood were sampled for analysis of drug concentrations.

#### Acute treatment with haloperidol

C57BL/6 male mice (age 10–16 wk) were injected intraperitoneally with a single dose of haloperidol (0.2 mg/kg) or saline vehicle. 45 min later, the animals were sacrificed, and the hippocampus and prefrontal cortex were extracted for protein analysis.

### Design of the Chronic Stress and Chronic PAR Treatment Experiment

We analyzed the ability of 51KO mice to recover from CSDS and the effects of chronic PAR treatment during a 3-wk recovery period. Initially, wild-type and 51KO mice were split into 2×2 groups (control wild-type, control 51KO, defeat wild-type, defeat 51KO) and subjected to the chronic stress procedure described below. After cessation of the stressor, groups were subdivided into vehicle-treated and PAR-treated animals, resulting in eight groups (vehicle: control wild-type, control 51KO, defeat wild-type, defeat 51KO; PAR: control wild-type, control 51KO, defeat wild-type, defeat 51KO; *n* = 7–13 per group). The treatment phase lasted 3 wk. The behavioral tests took place during the last week of the PAR treatment. PAR (GlaxoSmithKline) was administered via drinking water at a daily dose of ∼20 mg/kg as described previously [39].

### Chronic Stress Procedure and Physiological Parameters

The CSDS paradigm lasted for 21 d and was conducted as described previously [40]. Briefly, the experimental mice were introduced into the home cage (45 cm×25 cm) of a dominant resident mouse and defeated shortly after. When the defeat was achieved, the animals were separated by a wire mesh, preventing physical but allowing sensory contact for 24 h. Each day, stressed animals were defeated by another unfamiliar dominant resident mouse in order to exclude a repeated encounter throughout the experiment. The daily defeat was performed between 11:00 and 16:00 h; varying starting times reduced the predictability of the stressor and therefore minimized a potential habituation effect. Experimental mice were always defeated by resident males during the entire stress period. Control mice were housed in their home cages during the course of the experiment. Both stress and control animals were handled daily during the stress procedure; body weight for all mice was assessed at the beginning of the experiment, after the cessation of the stress period, and on the day of killing. Animals that underwent the stress procedure were subsequently single housed in standard cages.

### Chronic Paroxetine Treatment

PAR (GlaxoSmithKline) was administered via drinking water as described previously [39]. Briefly, the PAR solution was diluted in tap water to a final concentration of 0.16 mg/ml. With average water consumption of 5 ml/mouse/day, the daily dose of PAR was ∼20 mg/kg body weight. Fluid intake was monitored daily, and the variation of fluid intake was found to be <10% over the course of the experiment.

### Behavioral Analyses

The behavioral tests were carried out between 08:30 and 12:30 h in the same room in which the mice were housed. The testing order was as follows: open-field test, social avoidance test, FST, and acute stress response test. All tests were analyzed using an automated videotracking system (Anymaze 4.20, Stoelting). All animals underwent the same testing battery in the same order. To minimize possible carryover effects of the different behavioral tests, the sequence of tests was arranged from the least stressful to the most stressful, with a minimum of 24 h between tests [41].

#### Open-field test

The open-field test was performed to investigate locomotion differences. Testing was carried out in an empty open-field arena (50 cm×50 cm×50 cm) made of gray polyvinyl chloride (PVC), which was evenly illuminated with 15 Lux. The low illumination of the open-field arena was chosen to specifically investigate locomotion behavior and not create an aversive center region that may induce anxiety-related behavior. Testing time was 10 min, and the main parameter of interest was the total distance travelled.

#### Social avoidance test

The social avoidance test was performed as described previously [42]. Briefly, animals were allowed to explore the open-field arena for 2.5 min with an empty wire mesh cage placed at one side of the apparatus. In a second stage, the animals were confronted with an unfamiliar CD1 resident mouse in the wire mesh cage for another 2.5 min. The ratio between the time in the interaction zone of the no-target trial and the time in the interaction zone of the target trial serves as a marker for disturbed social behavior associated with depressive disorders. Animals that did not explore the interaction zone at all were excluded from the analysis (excluded: wild-type control/vehicle, 1; 51KO control/vehicle, 1; wild-type control/PAR, 2; 51KO control/PAR, 1; wild-type stress/vehicle, 1; 51KO stress/vehicle, 1; wild-type stress/PAR, 1). The FST was performed one day after the social avoidance test.

#### Forced swim test

In the FST, each mouse was put into a 2-l glass beaker (diameter: 13 cm, height: 24 cm) filled with tap water (21±1°C) to a height of 15 cm, so that the mouse could not touch the bottom with its hind paws or tail. Testing duration was 5 min. Time spent immobile (floating) and time spent struggling was scored by an experienced observer, blind to treatment or condition of the animals.

#### Acute stress response test

The FST also served as an acute stressor in order to determine the stress response by measuring corticosterone plasma concentrations. After the FST, all mice were towel-dried and placed into their home cage to recover from the acute stressor. Blood samples were taken by tail cut [43] 30 min (stress response) and 90 min (stress recovery) after the onset of the FST. Samples were collected in 1.5-ml EDTA-coated microcentrifuge tubes (Kabe Labortechnik). All blood samples were kept on ice and later centrifuged at 8,000 rpm at 4°C for 15 min. Plasma was transferred to new, labeled tubes and stored at −20°C until determination of corticosterone by radioimmunoassay (MP Biomedicals; sensitivity 12.5 ng/ml).

### Sampling Procedure

24 h after the last defeat, blood samples were taken by tail cuts, in order to assess the plasma corticosterone levels immediately after the chronic stress paradigm. 3 wk after the last defeat, thus following 3 wk of treatment, all animals were killed by decapitation following quick anesthesia by isoflurane at the end of the experiment. Basal trunk blood samples were processed as described above. Adrenal and thymus glands were removed, dissected from fat, and weighed. Prefrontal cortex and hippocampus were prepared for protein analysis.

The locations of brain regions were determined according to a mouse brain atlas and were dissected using a mouse brain slicer that allowed the dissection of discrete regions of the brain. The prefrontal cortex dissections included prelimbic, infralimbic, and orbitofrontal areas; the hippocampal dissections encompassed dorsal and ventral subdivisions.

### Determination of Drug Concentrations in Rodent Blood and Brain

Tissue extraction and determination of drug concentrations by high-performance liquid chromatography was performed as described previously [44],[45].

### Analysis of GFP-LC3B in Astrocytes

2×10^6^ cells were transfected with 2 µg of GFP-LC3B-expressing plasmid or the respective cloning vector using the Amaxa Nucleofector. Cells were grown and treated where indicated for 72 h and analyzed by fluorescence microscopy. At least 20 cells were counted.

### Human Samples

#### Healthy individuals

Protein-protein correlations were determined in extracts from peripheral blood mononuclear cells (PBMCs) from 21 healthy men (age 25.8 y, standard deviation [SD] 2.7 y). Of these, 14 individuals received 1.5 mg of dexamethasone (DEX), and protein expression changes in PBMCs were determined 6 h later. FKBP51-dependent antidepressant effects were evaluated in cultivated PBMCs from another 20 healthy men (age 34.8 y, SD 6.9 y).

#### Patients with depression

51 consecutive participants (26 women, 25 men; age 49.0 y, SD 14.7 y) of the Munich Antidepressant Response Signature (MARS) project were included for protein expression analyses, PBMC cultivation, and ex vivo treatment with antidepressants. The MARS project is an open-label prospective clinical study conducted at the hospital of the Max Planck Institute of Psychiatry and collaborating hospitals in southern Bavaria and Switzerland to evaluate typical depression courses under standard antidepressant treatment [46]. Patients were diagnosed according to DSM-IV criteria [47]. Patients suffered from major depressive disorder (single episode, *n* = 13; recurrent, *n* = 32) or from a depressive episode within a bipolar disorder (*n* = 6). Depression severity was assessed using the 21-item Hamilton Depression Rating Scale (HDRS) [48]; patients had an average baseline score of 23.8 (SD 5.6), indicating severe depression. Patients received antidepressant treatment in a naturalistic study setting [46]. Antidepressant treatment outcome was evaluated as percent change in HDRS score between baseline and after 6 wk of treatment. The resulting variable was approximately normally distributed with mean = 52.9 (SD 32.7) with a Kolmogorov-Smirnov test suggesting no deviation from the theoretical distribution (*p* = 0.715). Fasting venous blood samples for extraction of PBMCs were drawn from patients between 08:00 and 09:00 h within 5 d after admission. Further clinical characteristics are summarized in Tables 1 and 2.

**Table 1:** Demographic and clinical characteristics of the MARS patient sample.

Characteristic	MARS Patients (*n* = 51)
**Gender, percent female**	26 (51%)
**Age, mean ± SD**	49.0±14.7 y
**Diagnoses, number (percent)**	
Major depressive disorder— single episode	13 (25%)
Major depressive disorder—recurrent	32 (63%)
Bipolar depression	6 (12%)
**Duration of current episode, MD (IQR)**	19 (8; 52) wk; range: 2–450 wk
**Number of previous episodes, MD (IQR)**	1 (0; 3) episodes; range: 0–20
**HDRS at admission, mean ± SD**	23.8±5.6

Because of a non-normal distribution of duration of the current episode and number of previous episodes, the median, the interquartile range, and the total range are reported for these two variables.

IQR, interquartile range (25% and 75% percentiles); MD, median.

**Table 2:** Regression model predicting cortisol response in the first and second dexamethasone/corticotropin releasing hormone test (in terms of total area under the curve) using gender, age, dexamethasone-suppressed baseline cortisol, and baseline copeptin as predictor variables.

Variable	First DEX/CRH Test (*n* = 122)		Second DEX/CRH Test (*n* = 120)	
	Beta	*p*-Value	Beta	*p*-Value
Gender	0.11	0.054	**0.14**	**0.025**
Age	−0.03	0.615	−0.04	0.546
Baseline cortisol	**0.78**	**<0.001**	**0.61**	**<0.001**
Baseline copeptin	**0.15**	**0.008**	**0.31**	**<0.001**

Baseline values have been collected under the suppressive effects of DEX, but prior to stimulation with 100 µg of human corticotropin releasing hormone. Values with *p*<0.05 are in bold.

CRH, corticotropin releasing hormone.

### Preparation and Treatment of Human Peripheral Blood Mononuclear Cells

Blood of healthy men and of patients with depression was collected via venipuncture, diluted with PBS, carefully loaded on Biocoll solution (BioChrom, L6113), and centrifuged at 800 *g* for 20 min (brakeless running down). PBMCs were enriched by selecting the interphase of the Biocoll gradient. PBMCs of the interphase were washed two times with ice-cold PBS. PBMCs were resuspended in RPMI and plated at 4×10^5^ cells/cm^2^. After recovery for 6 h, cells were treated with 888 nM (120 ng/ml) AMI, 1,695 nM (500 ng/ml) fluoxetine (FLX), or 365 nM (120 ng/ml) PAR. Concentrations had been chosen to match therapeutic concentrations in the serum according to the consensus guidelines for therapeutic drug monitoring in psychiatry [49].

### Ethics Statement

Approval for the MARS project was received from the ethics committee in charge (submission number 318/00, ethics committee of the Medical Faculty at Ludwig Maximilian University of Munich, Germany), and participants gave oral and written consent before study inclusion.

The animal experiments were carried out in accordance with European Communities' Council Directive 2010/63/EU. All efforts were made to minimize animal suffering during the experiments; all experiments were based on the 3R principles: reduction, refinement, replacement. The protocols were approved by the Committee for the Care and Use of Laboratory Animals of the Government of Upper Bavaria, Germany.

### Statistical Analysis

The data presented were analyzed using the commercially available software SPSS 16.0. When two groups were compared, the Student's *t*-test was applied. For three or more group comparisons, one-way, two-way, or three-way analysis of variance (ANOVA) was performed, followed by Tukey's post-hoc test, as appropriate. All ANOVA *F-* and *p*-values are reported in Table S1; significant results of the contrast tests are indicated by asterisks in the figures. Protein-protein associations were analyzed using Pearson correlations. In the clinical sample, associations between human protein expression and antidepressant treatment outcome as well as protein-protein interactions were evaluated with Pearson correlations after correction for the effects of age and gender (partial correlation). *p*<0.05 was considered significant.

## Results

### FKBP51-Dependent Effects of Paroxetine and Amitriptyline on Behavior in FKBP51 Knockout Mice and Identification of FKBP51-Directed Pathways of Autophagy

**Figure 1:**
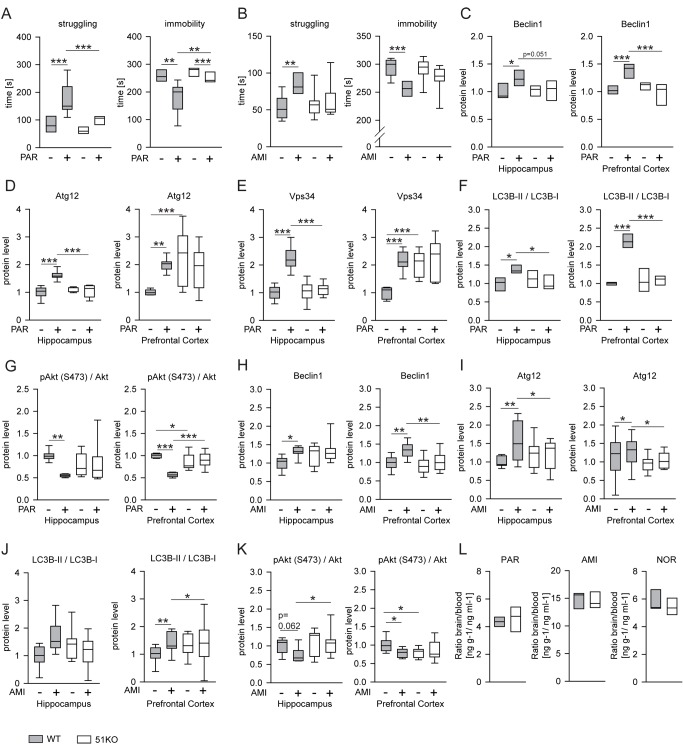
FKBP51 is required for acute effects of paroxetine and amitriptyline on behavior and autophagic pathways. (A and B) Two batches of 51KO and wild-type mice were treated with PAR (10 mg/kg) or vehicle (A), or with AMI (10 mg/kg) or vehicle (B) and subjected to the FST (*n* = 7–9 per group). Graphs show the times of immobility and struggling. (C–G) 51KO and wild-type mice were treated with PAR (10 mg/kg) or vehicle and sacrificed 45 min later. The levels of the indicated proteins were determined in extracts of the hippocampus and the prefrontal cortex from 9–11 animals per group. (H–K) Similar experiments were performed with AMI (10 mg/kg) instead of PAR. (L) Deletion of FKBP51 does not change blood-brain barrier function. 51KO and wild-type mice were treated with AMI (10 mg/kg, *n* = 5 for both wild-type and 51KO mice) or PAR (10 mg/kg, *n* = 4 for wild-type mice, *n* = 3 for 51KO mice) and sacrificed 45 min later. Levels of PAR, AMI, and the active AMI metabolite nortriptyline (NOR) were determined in blood and brain. Graphs display the ratio of the concentration in the brain (nanograms of drug per gram of brain tissue) and the concentrations in the plasma (nanograms of drug per milliliter of plasma). *p<0.05; **p<0.01; ***p<0.001. Statistical parameters in Table S1.

To assess whether FKBP51 modulates antidepressant response in mice, we treated wild-type and 51KO mice with an acute dose (10 mg/kg, as described previously [50]–[52]) of the antidepressants PAR or AMI. After 45 min, we subjected the mice to the FST as a well-established test for animals' response to antidepressants. PAR-treated wild-type mice spent less time floating (immobility) compared to vehicle-treated wild-type mice (*p* = 0.001), thus exhibiting the expected response to antidepressants. In contrast, the effect of PAR was significantly less pronounced in 51KO mice (wild-type/PAR versus 51KO/PAR, *p* = 0.001) (Figure 1A). Similarly, PAR treatment led to an increase in the time spent struggling in wild-type mice (*p* = 0.001). This effect of PAR was significantly attenuated in 51KO mice (wild-type/PAR versus 51KO/PAR, *p* = 0.001) (Figure 1A). Treatment with AMI produced similar results, i.e., deletion of FKBP51 entailed loss of antidepressant effects on struggling and floating times (Figure 1B).

To evaluate molecular effects of antidepressants that may depend on FKBP51 and parallel the behavioral phenotype, we analyzed autophagy markers in protein extracts from the hippocampus and prefrontal cortex after antidepressant injection (10 mg/kg). In wild-type mice, PAR treatment increased the levels of Beclin1 (hippocampus, *p* = 0.005; prefrontal cortex, *p* = 0.001), Atg12 (hippocampus, *p* = 0.001; prefrontal cortex, *p* = 0.004), and Vps34 (hippocampus, *p* = 0.001; prefrontal cortex, *p* = 0.001); increased the ratio of LC3B-II/I (hippocampus, *p* = 0.005; prefrontal cortex, *p* = 0.001); and evoked dephosphorylation of the kinase Akt (hippocampus, *p* = 0.002; prefrontal cortex, *p* = 0.001; phosphorylation at S473 in Akt1 represents the activated form [pAkt^S473^]); in contrast, 51KO mice showed no alterations in protein levels of autophagy markers or pAkt^S473^ in either brain region after PAR treatment (Figures 1C–1G and S1A). Similar results were obtained with AMI-treated mice, where the antidepressant effects on markers of autophagy were largely abolished in 51KO mice (Figures 1H–1K and S1B).

We considered the possibility that FKBP51 may act as an inhibitor of the blood-brain barrier, and its deletion may thus lead to a more efficient removal of antidepressants from the brain. We determined the levels of PAR, AMI, and nortriptyline—an active metabolite of AMI—in the blood and in the brain 45 min after injection of PAR or AMI in wild-type and 51KO mice. No indications of enhanced blood-brain barrier function could be detected for any of the drugs (Figure 1L).

**Figure 2:**
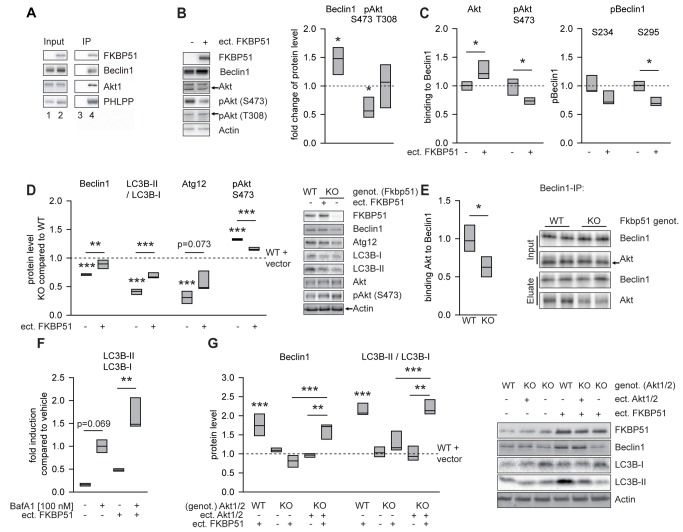
FKBP51 directs autophagic pathways. (A) FKBP51 interacts with Beclin1, Akt, and PHLPP. HEK cells were transfected with vector control (lanes 1 and 3) or a FLAG-tagged FKBP51-expressing plasmid (lanes 2 and 4) and lysed 72 h later. After immunoprecipitation (IP) of protein complexes using a FLAG antibody, input (lanes 1 and 2) and (co)precipitated (lanes 3 and 4) proteins were visualized by Western blotting. (B) Change of total Beclin1, pAkt^S473^, and pAkt^T308^ upon expression of FKBP51. Representative Western blots on the right. FKBP51 was detected by an antibody directed against its FLAG tag. (C) FKBP51 interacts preferentially with dephosphorylated Akt and promotes Akt dephosphorylation. Graphs display quantification of pBeclin1 (S234 and S295) and Beclin1-Akt/pAkt^S473^ interaction after Beclin1 immunoprecipitation from HEK cells transfected with FKBP51/52 (details in Figure S2). (D) Comparison of the endogenous protein levels of effects of Beclin1, LC3B-II/I, Atg12, and pAkt^S473^ levels in wild-type MEFs, 51KO MEFs, and 51KO MEFs transfected with FKBP51 or vector control. Graphs show the relative expression, with the levels of wild-type cells transfected with vector control set to 1 (dashed line). Representative Western blots on the right. (E) Quantification of Beclin1-Akt interaction after Beclin1 immunoprecipitation in brain extracts from wild-type and 51KO mice. Graph represents the results from five independent experiments. Representative Western blots on the right. (F) FKBP51 enhances autophagic flux. Cortical rat astrocytes were transfected with FKBP51 or vector and treated with bafilomycin A_1_ (BafA1) as indicated, and the levels of LCB3-II/I were determined. (G) Cellular effects of FKBP51 on autophagic markers require Akt1 and/or Akt2. The protein levels of Beclin1 and LC3B-II/I were evaluated in wild-type MEFs, Akt1/2KO MEFs, and Akt1/2KO MEFs transfected with Akt1- and Akt2-expressing plasmids. Graphs display the relative expression of three different experiments; expression in wild-type vector-transfected MEFs was set to 1 (dashed line). Representative Western blots on the right. *p<0.05; **p<0.01; ***p<0.001. See Table S1 for statistical details. ect., ectopic; genot., genotype; KO, 51KO; WT, wild-type.

The results in 51KO mice suggest a role of FKBP51 in autophagic events, which we further evaluated using biochemical and cell biological methods. Because FKBP51 has been reported to interact with Akt [53], which in turn regulates Beclin1 [19], we initially tested whether FKBP51 interacts with Beclin1. FLAG-tagged FKBP51 was expressed in HEK cells to assess its interaction with the autophagy initiator Beclin1 by CoIP. FKBP51 displayed binding to Beclin1 (Figure 2A), in addition to the previously reported interaction with Akt and the phosphatase PHLPP [53].

A direct effect of Akt on Beclin1 has been identified as an important mechanism blocking autophagy [19]. Thus, we next tested whether FKBP51 affects the interaction between Akt and Beclin1. Increasing the levels of FKBP51 by transient transfection in HEK cells led to higher overall levels of Beclin1 (*p* = 0.031; Figures 2B and S2A) and to higher amounts of Akt co-precipitating with Beclin1 (*p* = 0.036, normalized to precipitated Beclin1; Figures 2C and S2B). In addition, FKBP51 lowered the amounts of pAkt^S473^ interacting with Beclin1 (*p* = 0.043; Figures 2C and S2B). Furthermore, FKBP51 increased the levels of autophagy promoting dephosphorylated Beclin1 (*p* = 0.011 for S295; Figures 2C and S2B). The overall level of pAkt^T308^ (conferring basal activity) was unchanged upon expression of FKBP51, while the pAkt^S473^ levels were reduced (*p* = 0.020; Figures 2B and S2A). This is in line with previous findings and has been explained by the recruitment of the phosphatase PHLPP to Akt [53]. Together, these findings support a model in which FKBP51 acts 2-fold on Beclin1: first, by increasing the overall Beclin1 levels and, second, by recruiting the inactive (dephosphorylated by PHLPP) form of Akt^S473^ to Beclin1.

To evaluate whether FKBP51's effects on Beclin1 are accompanied by alterations of other autophagy markers, we compared 51KO and wild-type MEFs. MEFs lacking FKBP51 displayed reduced levels of Beclin1 and decreased levels of two other autophagy proteins, LC3B-II/I and Atg12 (Figure 2D). Consistent with the reduced levels of pAkt^S473^ observed in HEK cells upon ectopic expression of FKBP51 (Figures 2B and S2A), deletion of FKBP51 in MEF cells resulted in higher levels of pAkt^S473^ (*p* = 0.001; Figure 2D). These effects of FKBP51 gene deletion were attenuated by ectopic expression of FKBP51 in 51KO MEFs (Figure 2D).

To test whether the impact of FKBP51 on protein interaction between Beclin1 and Akt characterized in HEK cells (Figure 2B) is also relevant to the mouse brain, we performed immunoprecipitation using protein extracts of the brain from wild-type and 51KO mice. Abolition of FKBP51 significantly reduced the interaction between Akt and Beclin1 (*p* = 0.011; Figure 2E).

While LC3B-II/I in itself is often used as a marker of autophagy, its increase per se is not proof of functional autophagic flux; in contrast, this increase may also indicate a block of autophagic flux [54]. Therefore, to test the possibility that FKBP51 may actually block autophagy at terminal stages, we analyzed the effect of FKBP51 in primary astrocytes as a model of neural cells in the presence and absence of bafilomycin A_1_, which is a V-ATPase inhibitor causing an increase in lysosomal pH, thereby blocking autophagic flux [54]. Ectopic expression of FKBP51 also increased LC3B-II/I in the presence of bafilomycin A_1_ (*p* = 0.003; Figures 2F and S3), indicating that FKBP51 not only leads to increases in early markers of autophagy, but also increases, at least partially, autophagic flux [54]. Together, the data corroborate a stimulatory role of FKBP51 on autophagy.

Given that FKBP51 acts on a variety of intracellular proteins, we used Akt1/2KO MEFs to test whether FKBP51's impact on pAkt^S473^ was required for its influence on autophagic markers. In wild-type MEFs, transfection of FKBP51 increased the levels of the autophagy markers Beclin1 (*p* = 0.001) and LC3B-II/I (*p* = 0.001; Figure 2G). These effects of FKBP51 were not observed in Akt1/2KO MEFs, but were restored after reintroducing Akt1 and Akt2 into Akt1/2KO MEFs by transient transfection (Figure 2G). Thus, the impact of FKBP51 on autophagy markers requires Akt1 and/or Akt2.

### Cellular Effects of Antidepressants on Autophagic Markers Depend on FKBP51

**Figure 3:**
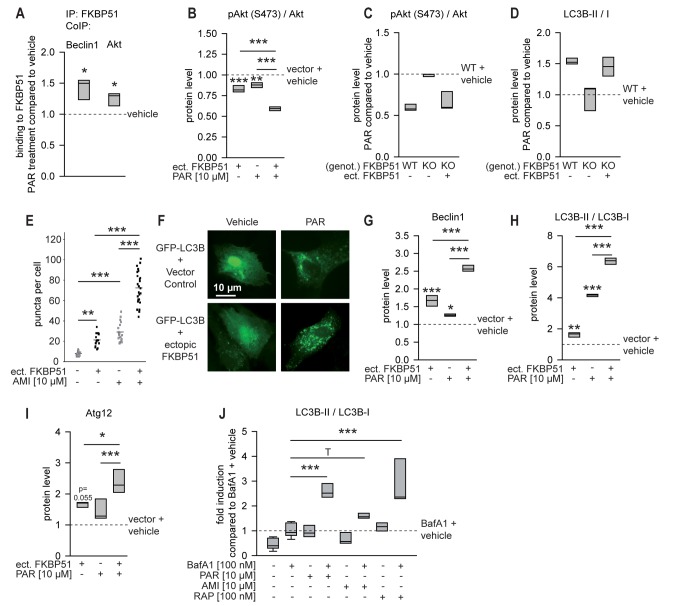
FKBP51 enhances cellular effects of antidepressants. (A) The interaction of FKBP51 with Beclin1 and Akt in HEK cells in the presence or absence of PAR (10 µM, 72 h) was analyzed by CoIP. (B) Primary rat cortical astrocytes were transfected with vector control or FKBP51 and treated with PAR (10 µM) for 72 h, and the pAkt^S473^/Akt ratio was determined. (C and D) Wild-type MEFs, 51KO MEFs, and 51KO MEFs transfected with FKBP51 or vector control were treated with PAR (10 µM) for 72 h; pAkt^S473^/Akt (C) and LC3B-II/I (D) ratios were determined. Protein levels in untreated wild-type MEFs were set to 1 (dashed line). (E) Primary astrocytes were cotransfected with GFP-LC3B and vector control or FKBP51, and treated with PAR (10 µM) for 72 h. The number of GFP-LC3B-positive puncta/cell was determined in 15–25 randomly selected cells for each condition. (F) Representative fluorescence images of (E). (G–I) Primary astrocytes were transfected with vector control or FKBP51, and treated as in (B); Beclin1, LC3B-II/I, and Atg12 levels were determined. Protein levels of untreated vector-transfected cells were set to 1 (dashed line). (J) Antidepressants enhance autophagic flux. Rat cortical astrocytes were incubated with the drugs as indicated for 2 h, and the levels of LC3B-I, LC3B-II, and Actin were determined in protein extracts by Western blotting. The graph displays the median ratio of LC3B-II/I from three independent experiments. *p<0.05; **p<0.01; ***p<0.001. Statistical parameters in Table S1. BafA1, bafilomycin A_1_; ect., ectopic; genot., genotype; IP, immunoprecipitation; KO, 51KO; RAP, rapamycin; WT, wild-type.

To determine potential effects of antidepressants on the FKBP51-directed autophagic events characterized above, we tested whether PAR, AMI, or FLX impacted on FKBP51 heterocomplexes in HEK cells. Each antidepressant was used at 10 µM, the concentration that proved sufficient for induction of autophagy markers in cell culture (Figure S4 and [14]). Treatment with PAR increased the amount of Akt and Beclin1 co-precipitating with FKBP51 (Akt, *p* = 0.014; Beclin1, *p* = 0.012; Figures 3A and S5A). Similar effects were observed with AMI, while FLX showed no effect (Figure S5A).

Both a decrease and an increase of pAkt^S473^ induced by antidepressants have been reported to date [55],[56]. To test the impact of antidepressants on pAkt^S473^ in neural cells, we used rat primary astrocytes. PAR led to decreased levels of pAkt^S473^ (*p*<0.001; Figures 3B and S5B); however, the decrease was not significant for AMI and FLX (Figures S5B and S5C). The antidepressant effect was stronger when FKBP51 was transfected into astrocytes (significant for PAR).

The importance of FKBP51 for antidepressant effects on components of the autophagy pathway was further evaluated in 51KO and Akt1/2KO MEFs. PAR, AMI, and FLX reduced pAkt^S473^ in wild-type MEFs, but not in 51KO MEFs; ectopic expression of FKBP51 in 51KO MEFs restored the effect of antidepressants on pAkt^S473^ (Figures 3C, S5D, and S5E). The autophagy marker LC3B-II/I was elevated upon antidepressant treatment in wild-type MEF cells (Figures 3D, S5D, and S5F), as reported previously for astrocytes [14]. This antidepressant effect was blunted in 51KO MEFs, and was restored when FKBP51 was ectopically expressed in these cells. In Akt1/2KO MEFs, antidepressant exposure did not induce changes in the ratio of LC3B-II/I (Figure S6A and S6B). When Akt1 and Akt2 were reintroduced by ectopic expression, the effects of antidepressants re-emerged.

The shift of LC3B from cytosolic to autophagosomal compartments is also a major hallmark of autophagy. The influence of FKBP51 on the capability of antidepressants to change the distribution of recombinant GFP-LC3B was monitored in primary cortical astrocytes. Ectopically expressed FKBP51 enhanced antidepressant-induced clustering of GFP-LC3B (*p* = 0.001; Figures 3E, 3F, S6C, and S6D). In the absence of antidepressants, FKBP51 triggered the formation of GFP-LC3B-positive puncta to a lesser extent. Further, FKBP51 enhanced the ability of antidepressants to induce conversion from LC3B-I to LC3B-II and to elevate Beclin1 and Atg12 levels in primary astrocytes (Figures 3G–3I, S5B, and S6E; *p* = 0.001 for Beclin1 and PAR, *p* = 0.001 for LC3B-II/I and PAR, *p* = 0.010 for Atg12 and PAR). Collectively, these data support the conclusion that the cellular antidepressant effects on autophagy pathways depend on FKBP51.

Although antidepressant exposure in cell culture is typically applied for 1–4 d, we hypothesized that short-term treatment for 45 min would also elicit autophagic makers, mirroring the 45-min acute treatment duration design in mice. Treatment of primary astrocytes revealed that PAR, AMI, and partly also FLX evoked a response of the autophagic markers Beclin1, Atg12, pAkt, and LC3B-II/I after 45 min of treatment (Figure S7).

We also tested whether the antidepressants actually affected autophagic flux under our experimental conditions. We found that antidepressants increased LC3B-II/I in the presence of bafilomycin A_1_ (*p* = 0.001 for both PAR and AMI; Figures 3J and S8), indicating at least some increase in autophagic flux [54]. The influence of FKBP51 on antidepressant effects was also evaluated in rat primary neurons. We found that FKBP51 and PAR also synergize in neurons to enhance Beclin1 protein levels (Figure S9).

The dissociative anesthetic ketamine and the tropane alkaloid scopolamine have been proposed as fast-acting antidepressants [57],[58]. In addition, they have been linked to the activity of the kinase mTOR, which is also a known inhibitor of autophagy. Therefore, we tested the potential effect of ketamine and scopolamine on autophagy markers, and additionally included the neuroleptic haloperidol as a non-antidepressant. Mice (*n* = 6) were intraperitoneally injected with drug or vehicle and sacrificed 45 min later for protein analysis of hippocampus and prefrontal cortex. None of the drugs induced markers of autophagy; scopolamine somewhat reduced the levels of Beclin1 (*p* = 0.005 at 1 µM, *p* = 0.001 at 3 µM; Figure S10), consistent with its reported activation of mTOR [58].

### The Effects of Chronic Paroxetine Treatment on Behavior and Autophagic Pathways in Mice Depend on FKBP51

**Figure 4:**
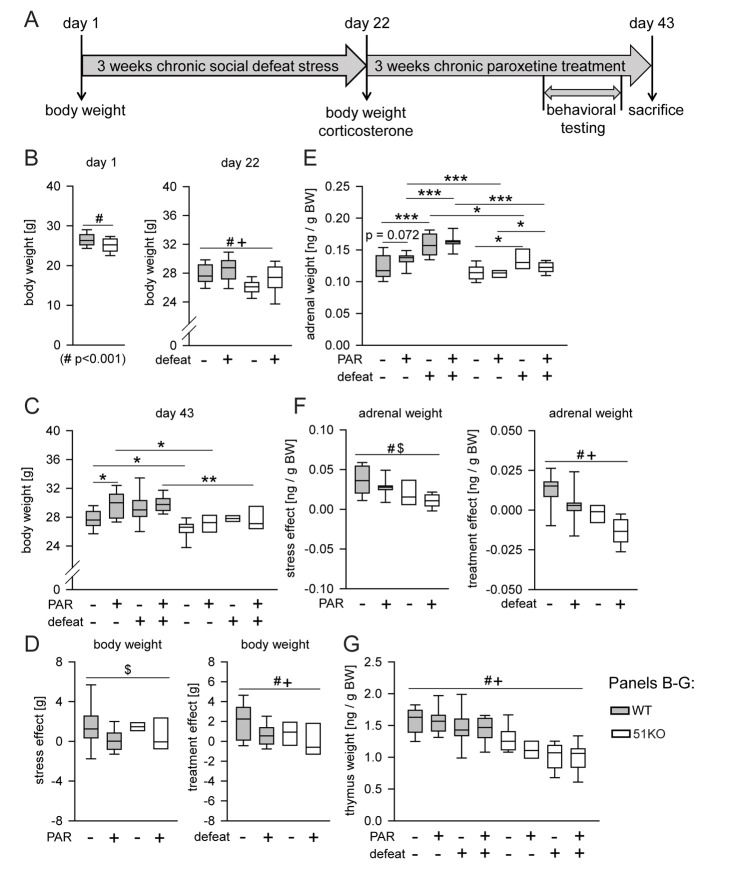
Deletion of FKBP51 diminishes the physiological effects of chronic stress and chronic PAR treatment. (A) Time course of the experiment. Body weight was assessed on day 1, day 22, and day 43. Basal corticosterone was measured on day 22 and day 43. The behavioral testing (open-field test, social avoidance test, and FST) and the stress response test (corticosterone samples 30 min and 90 after the FST) were performed in the last week of the treatment phase. (B–G) Weights of body, thymus, and adrenal glands. When the three-way ANOVA indicated an interaction effect (*p*<0.1), genotype-dependent stress and treatment effects were isolated by normalizing the data to either unstressed controls or vehicle treatment. (B) Body weight was significantly reduced in 51KO mice before the start of the first defeat. At day 22, significant main effects for condition and genotype were observed: CSDS induced an increase of body weight in both wild-type and 51KO mice, while 51KO mice still showed reduced body weight compared to wild-type mice. (C and D) Stress-induced body weight increase was still present after 3 wk of recovery. PAR-induced increase of body weight was most pronounced under control conditions. 51KO mice were less affected by PAR-induced body weight increase. (E and F) 51KO mice showed overall reduced adrenal gland weight compared to wild-type mice. Adrenal gland weight was significantly increased in stressed animals. The stress effect was less pronounced in 51KO mice than in wild-type animals. PAR led to adrenal gland enlargement in wild-type mice and reduction in 51KO mice. (G) Thymus size was significantly reduced following CSDS. 51KO mice demonstrated significantly lower thymus weight than wild-type mice. Asterisks indicate significant result for planned contrast test for main genotype effect (#), main treatment effect ($), or main condition effect (+): *p<0.05; **p<0.01; ***p<0.001. See Table S1 for all statistical parameters. BW, body weight; WT, wild type.

To more closely mimic the clinical situation, we also assessed the effect of FKBP51 deletion in mice after chronic PAR or vehicle treatment; in addition, we applied CSDS, as it is suggested to model some endophenotypes of depression [28]. Mice were subjected to 3 wk of CSDS, followed by 3 wk of PAR treatment, with behavioral tests during the last week (Figure 4A).

The results determined from body weight, adrenal weight, thymus weight, and corticosterone levels show that FKBP51 shapes the long-lasting physiological and neuroendocrine effects of CSDS (Figures 4 and S11), consistent with our previous findings on the immediate effects of FKBP51 on chronic stress [5].

On the day of sacrifice (day 43, after 3 wk of treatment), we found a main genotype effect (*p*<0.0001) (for statistical details see Table S1) and a condition×treatment interaction (*p* = 0.074) for body weight (Figure 4C). In order to isolate genotype-dependent stress and treatment effects, we normalized the data to either unstressed controls or vehicle treatment. First, we normalized all stressed groups by assessing the difference in body weight compared to their corresponding unstressed control group of the same genotype and treatment. We found a significant treatment effect (*p* = 0.024): stress-induced increase in body weight was significantly more pronounced in vehicle-treated mice than in PAR-treated mice (Figure 4D, left). To isolate stress effects as well as genotype-dependent treatment effects, we then normalized all PAR-treated groups by assessing the difference in body weight compared to their corresponding vehicle treated control groups of the same genotype and condition. Two-way ANOVA revealed a significant condition (*p* = 0.02) and genotype effect (*p* = 0.042). PAR-induced body weight increase was significantly enhanced in mice under basal conditions compared to stressed animals. Interestingly, 51KO mice were significantly less affected by PAR-induced body weight increase than wild-type mice (Figure 4D, right).

**Figure 5:**
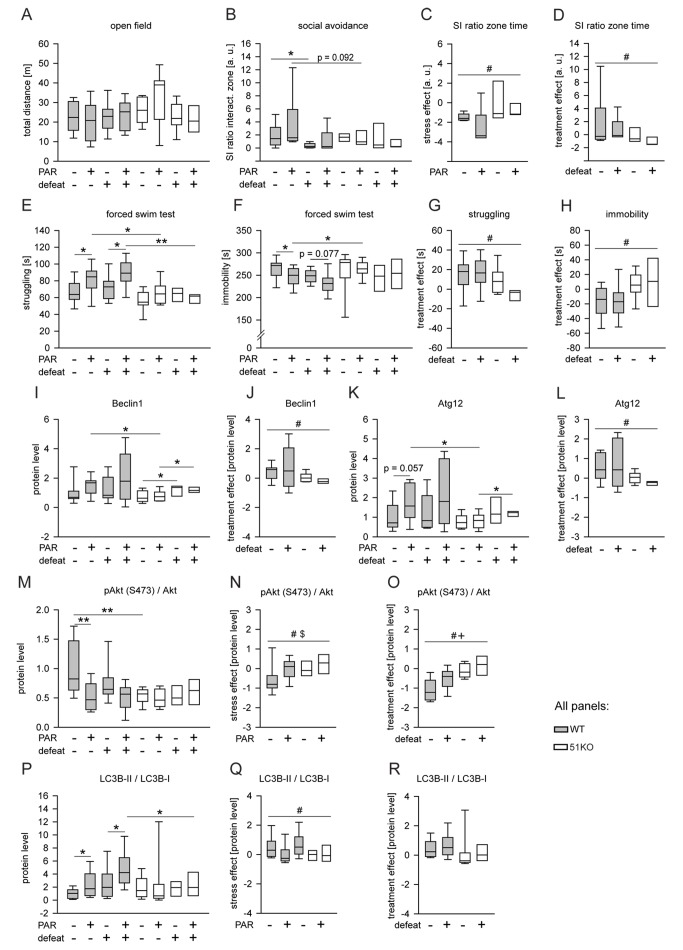
FKBP51 is required for the chronic effects of PAR on behavior and autophagic pathways. Wild-type and 51KO mice were subjected to CSDS and subsequent chronic PAR treatment resulting in eight experimental groups (*n* = 8–12 per group) that were analyzed for behavior and autophagy marker protein levels. When the three-way ANOVA indicated an interaction effect (*p*<0.1), genotype-dependent stress and treatment effects were isolated by normalizing the data to either unstressed controls or vehicle treatment. (A) General locomotion in the open-field arena was independent of genotype, condition, or treatment of the mice. (B–D) Deletion of FKBP51 abolished the reaction to CSDS and to chronic PAR treatment in the social avoidance test. (E–H) Deletion of FKBP51 significantly reduced the effect of chronic PAR treatment in the FST. (I–R) Deletion of FKBP51 abolished the effects of chronic PAR treatment on the autophagy pathway markers Beclin1, Atg12, pAkt, and LC3B-II/I. Protein parameters were analyzed in hippocampal brain extracts. Asterisks indicate significant result for planned contrast test for main genotype effect (#), main treatment effect ($), or main condition effect (+): *p<0.05; **p<0.01. a.u., arbitrary units; SI zone, social interaction zone; WT, wild type.

To assess the behavioral effects of FKBP51 deletion on the actions of CSDS and chronic PAR treatment we used a social interaction paradigm in addition to the FST. Moreover, we analyzed general locomotion in an open-field test to control for potential confounding effects. The total distance traveled in the open-field test did not differ between genotype, condition, or treatment groups (Figure 5A). In the social avoidance test, deletion of FKBP51 resulted in loss of effects by CSDS and by chronic PAR treatment. Three-way ANOVA revealed a main treatment effect (*p* = 0.016), a genotype×condition interaction (*p* = 0.077), and a genotype×treatment interaction (*p* = 0.043) (Figure 5B). To isolate treatment effects and genotype-dependent stress effects, we normalized all stressed groups by assessing the difference in the social interaction ratio compared to their corresponding unstressed control group of the same genotype and treatment. We found that CSDS significantly reduced social behavior in wild-type mice, but not in 51KO mice (*p* = 0.003) (Figure 5C). To isolate stress effects and genotype-dependent treatment effects, we normalized all PAR-treated groups by assessing the difference in the social interaction ratio compared to their corresponding vehicle-treated control group of the same genotype and condition. PAR significantly enhanced social behavior in wild-type mice, but not in 51KO mice, both in CSDS-exposed and control animals (*p* = 0.024) (Figure 5D). In the FST, chronic PAR treatment was significantly less effective in enhancing struggling and reducing floating in FKBP51-deleted mice compared to wild-type mice. We observed a genotype×treatment interaction for the parameters struggling (*p* = 0.056) and floating (*p* = 0.032) (Figure 5E and 5F). To isolate stress effects and genotype-dependent treatment effects, we normalized all PAR-treated groups by assessing the difference in struggling and floating compared to their corresponding vehicle-treated control group of the same genotype and condition. In both parameters, 51KO mice were significantly less affected by PAR treatment than wild-type mice, independent of condition (struggling: *p* = 0.017; floating: *p* = 0.002) (Figure 5G and 5H).

Analysis of the protein parameters of autophagic pathways in the brains of mice exposed to CSDS and chronic PAR revealed a main genotype effect (*p* = 0.037), a main condition effect (*p* = 0.013), and a genotype×treatment interaction (*p* = 0.049) for Beclin1 (Figure 5I) and for Atg12 (Figure 5K). PAR increased Beclin1 (*p* = 0.016) (Figure 5J) and Atg12 (*p* = 0.008) (Figure 5L) in wild-type mice, but not in 51KO mice. For pAkt, a main genotype effect (*p* = 0.017), a main treatment effect (*p* = 0.008), and genotype×treatment interaction (*p* = 0.005) were observed (Figure 5M). CSDS and PAR elicited an effect (*p* = 0.049) on pAkt in wild-type mice, but not in 51KO mice (Figure 5N and 5O). For LC3B-II/I, a main treatment effect (*p* = 0.030) and genotype×condition interaction (*p* = 0.069) were found (Figure 5P). CSDS and PAR increased LC3B-II/I in wild-type mice, but not in 51KO mice (CSDS: *p* = 0.013; PAR: *p* = 0.080) (Figure 5Q and 5R).

The data from CSDS and chronic PAR treatment support the conclusion that FKBP51 also impacts on the effects of chronic PAR treatment.

### Human Studies

To translate the findings derived from cellular and animal experiments to humans, we used PBMCs from different cohorts. PBMCs from 21 healthy men (age 25.8 y, SD 2.7 y) were analyzed for protein-protein correlations. Of these healthy individuals, 14 received the synthetic glucocorticoid DEX to evaluate the link of FKBP51-directed pathways to stress. PBMCs from another 20 healthy men (age 34.8 y, SD 6.9 y) were cultivated to assess FKBP51-dependent antidepressant effects. The patient cohort consisted of 51 consecutive participants (26 women, 25 men; age 49.0 y, SD 14.7 y) of the MARS project [46]. Depression severity was assessed by using the 21-item HDRS [48]; patients had an average baseline score of 23.8 (SD 5.6), indicating severe depression. More details of the clinical sample are provided in Table 1.

### Effects of Antidepressants and of Dexamethasone in Blood Cells of Healthy Individuals Depend on FKBP51

**Figure 6:**
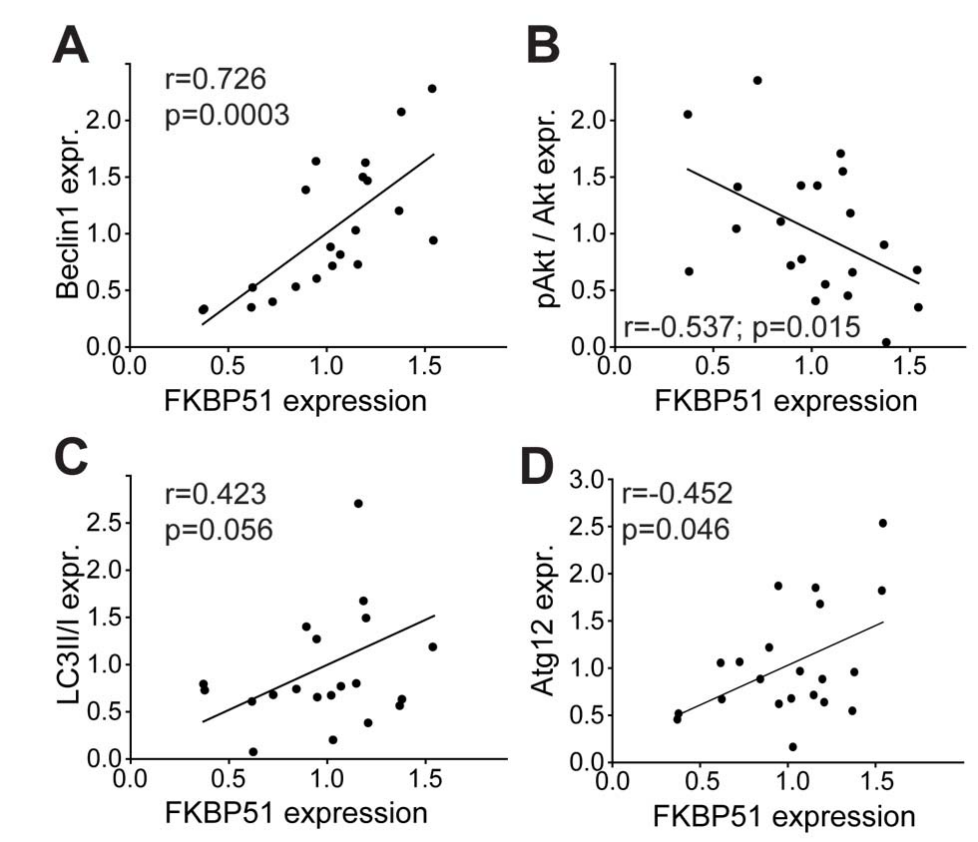
Correlation of FKBP51 with autophagy pathway components in human PBMCs. Protein levels of FKBP51 and Beclin1 (A), pAkt^S473^/Akt (B), LC3B-II/I (C), and Atg12 (D) in PBMCs from healthy men (*n* = 21). Each dot represents the levels of FKBP51 and the respective protein in the PBMCs from one individual. Average expression levels were set to 1.

Determination of basal FKBP51 expression in PBMCs derived from healthy men revealed marked variations (Figures 6 and S12A). The FKBP51 expression level was positively correlated with the expression of Beclin1 (Pearson correlation coefficient *r* = 0.726, *p* = 0.0003) and Atg12 (Pearson correlation coefficient *r* = 0.452, *p* = 0.046), and negatively correlated with the phosphorylation status of Akt^S473^ (Pearson correlation coefficient *r* = −0.537, *p* = 0.015); LC3B-II/I displayed a non-significant correlation with FKBP51 protein levels (Pearson correlation coefficient *r* = 0.423, *p* = 0.056) (Figure 6).

**Figure 7:**
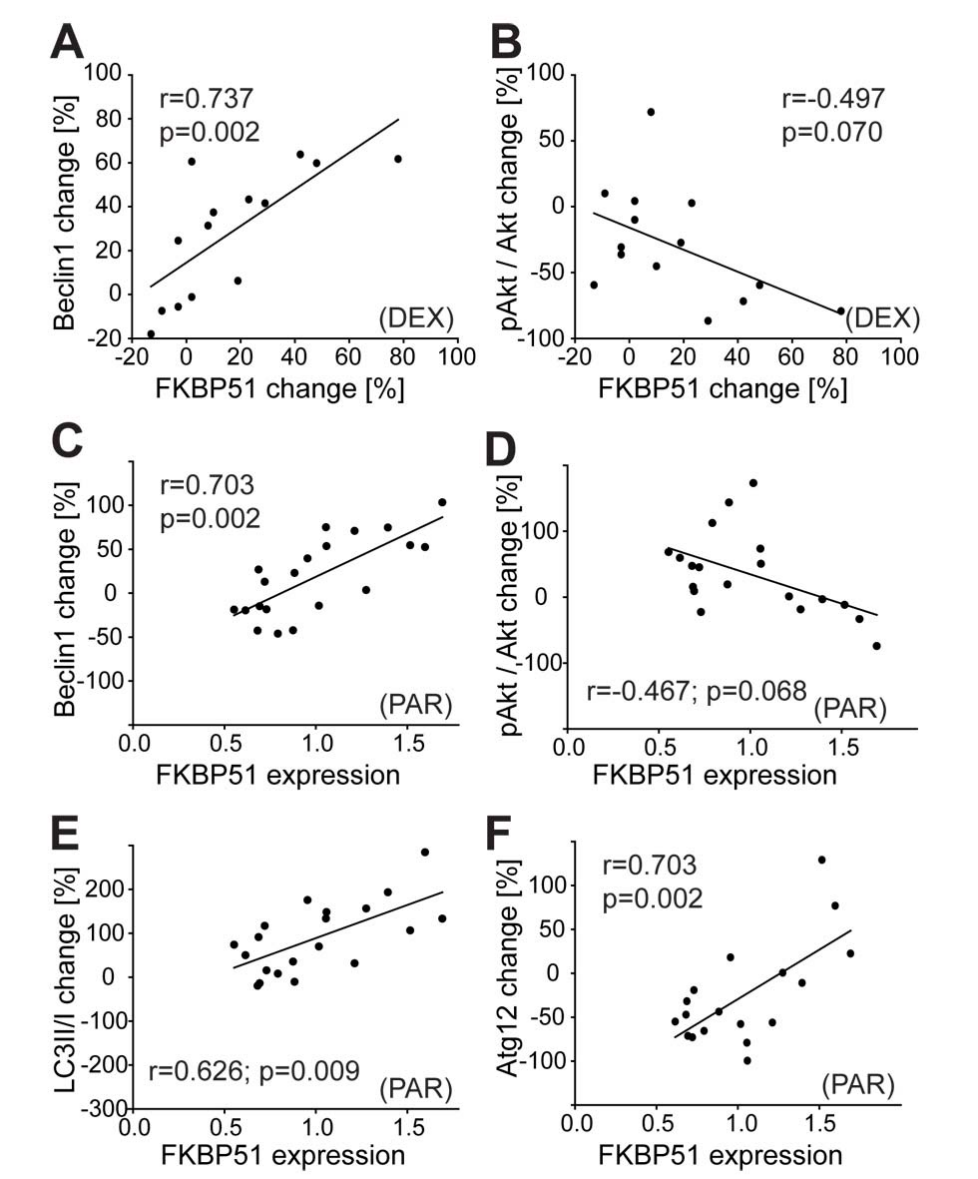
Effects of dexamethasone and of antidepressants on autophagic pathway components in human PBMCs correlate with FKBP51. (A and B) PBMCs were collected from healthy subjects (*n* = 14) before and 6 h after the intake of 1.5 mg of DEX, and proteins were analyzed. Each dot represents the DEX-induced change in FKBP51 and the expression change of the respective protein (Beclin 1 [A] and pAkt^S473^ [B]) in the PBMCs of one individual. (C–F) Levels of FKBP51 and Beclin1 (C), pAkt^S473^ (D), LC3B-II/I (E), and Atg12 (F) in PBMCs from healthy men (*n* = 21) cultivated ex vivo and treated with PAR (0.365 µM, 48 h). Plots depict protein changes upon treatment with antidepressant compared to vehicle-treated cells in correlation to FKBP51. Each dot represents the level of FKBP51 and the expression change of the respective protein in the PBMCs from one individual.

Since FKBP51 is a stress-responsive gene [3],[59] and glucocorticoids have been shown to induce autophagy [60], FKBP51 might also link stress to autophagy. We determined the expression of Beclin1 and FKBP51 in PBMCs from healthy men before and 6 h after administration of the synthetic glucocorticoid DEX (1.5 mg). There was a significant correlation between the induction of FKBP51 and the increase in Beclin1 expression (Pearson correlation coefficient *r* = 0.737, *p* = 0.002; Figures 7A and S12B), and a non-significant correlation between the induction of FKBP51 and the dephosphorylation of pAkt^S473^ (Pearson correlation coefficient *r* = −0.497, *p* = 0.07; Figures 7B and S12B). In an animal model, DEX affected levels of Beclin1, Vps34, and pAkt^S473^ in cortical astrocytes from wild-type mice, but not from 51KO mice (Figure S12C–S12F).

PBMCs from healthy men were also cultivated ex vivo and treated with AMI, FLX, or PAR for 48 h. To more closely mimic the clinically relevant in vivo conditions, drug concentrations were chosen according to actual consensus guidelines for therapeutic drug monitoring in psychiatry [49]. The extent of dephosphorylation of pAkt^S473^, change of LC3B-II/I ratio, and change in levels of Beclin1 and Atg12 in response to antidepressants correlated with the expression level of FKBP51 (Figures 7C–7F, S12G, and S12H; Pearson correlation coefficient for PAR in the case of Beclin 1, *r* = 0.703, *p* = 0.002; LC3B-II/I, *r* = 0.626, *p* = 0.009; pAkt^S473^, *r* = −0.467, *p* = 0.068; and Atg12, *r* = 0.665, *p* = 0.005). Higher expression of FKBP51 was associated with antidepressant-induced dephosphorylation of pAkt^S473^, and lower expression of FKBP51 was associated with antidepressant-enhanced pAkt^S473^. With increasing FKBP51 levels, a gradual shift from slightly inhibitory to stimulatory effects of antidepressants on Beclin1 expression was observed. Atg12 expression was not significantly changed by AMI or FLX. Taken together, FKBP51 is required for the effects of antidepressants and of DEX on autophagic pathways in humans.

### Clinical Improvement of Patients with Depression Is Predicted by Their Levels of Beclin1 and FKBP51 at Hospital Admission

**Figure 8:**
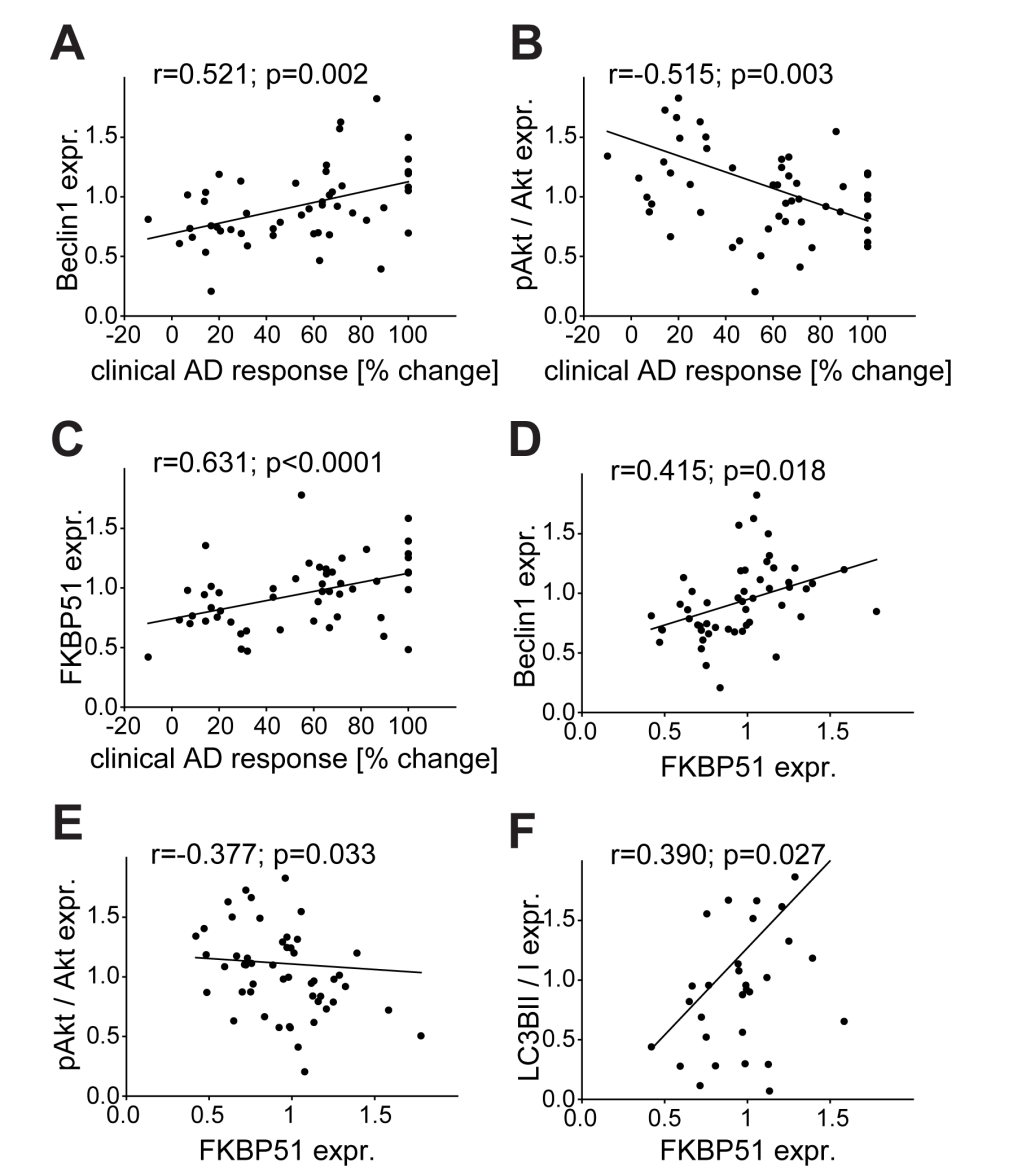
Correlation of the expression of FKBP51, pAkt, and Beclin1 with clinical antidepressant response, and of the autophagic markers Beclin1, pAkt, and LC3B-II/I with FKBP51. Beclin1, FKBP51, LC3B-II/I, and pAkt^S473^/Akt levels were determined in PBMCs from inpatients with depression at admission (*n* = 51). (A–C) Expression values of Beclin1 (A), pAkt^S473^/Akt (B), and FKPP51 (C) are correlated with the response to antidepressant (AD) treatment expressed as percent change in total score on the 21-item HDRS between admission and after 6 wk of treatment. (D–F) Expression values of the autophagy pathway markers Beclin1 (D), pAkt^S473^/Akt (E), and LC3B-II/I (F) are correlated with the expression levels of FKBP51.

To further evaluate the clinical relevance of our findings, we determined the expression levels of Beclin1, FKBP51, and pAkt^S473^ in PBMCs from inpatients (for demographic and clinical parameters see Methods and Ttables 1 and 2) with a current depressive episode at admission and compared them with the clinical treatment response. The clinical response after 6 wk of treatment showed statistically significant positive correlations with the levels of Beclin1 (Pearson correlation coefficient *r* = 0.521, *p* = 0.002) and FKBP51 (Pearson correlation coefficient *r* = 0.6311, *p*<0.0001), and a negative correlation with pAkt^S473^ (Pearson correlation coefficient *r* = −0.515, *p* = 0.003) (Figure 8A–8C). The protein analyses in the PBMCs from patients with depression also confirmed the correlation between the levels of autophagy markers and the expression levels of FKBP51 (Figure 8D–8F).

### Reactivity of Beclin1 and pAkt of Patient-Derived PBMCs to Antidepressant Treatment Ex Vivo Predicts Clinical Improvement

**Figure 9:**
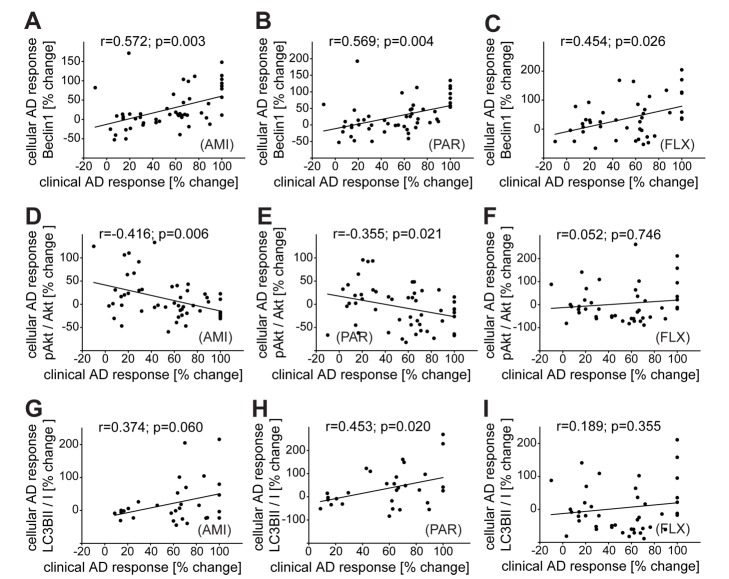
Correlation of clinical antidepressant response with the effects of antidepressants in PBMCs ex vivo. PBMCs from inpatients with depression were cultivated ex vivo and treated with PAR (0.365 µM), FLX (1,695 nM), or AMI (888 nM) for 48 h. The antidepressant-induced changes in expression of Beclin1 (A–C), pAkt^S473^/Akt (D–F), and LC3B-II/I (G–I) are correlated with the clinical response to antidepressant (AD) treatment expressed as percent change in total score on the 21-item HDRS between admission and after 6 wk of treatment. The collected PBMCs were not always sufficient to run all conditions or analyze all proteins: (A, B, D, and E): *n* = 51; (C): *n* = 43; (F): *n* = 42; (G): *n* = 30; (H): *n* = 31, (I): *n* = 27.

PBMCs were also collected from inpatients at admission, cultivated, and treated with AMI, FLX, or PAR ex vivo for 48 h. We observed a correlation between therapeutic outcome and the increase of Beclin1 levels in treated PBMCs for all three antidepressants (Pearson correlation coefficient in the case of PAR, *r* = 0.569, *p* = 0.004; AMI, *r* = 0.572, *p* = 0.003; FLX, *r* = 0.454, *p* = 0.024) (Figure 9A–9C). There was a significant negative correlation between therapeutic outcome and the change of pAkt^S473^ upon treatment with AMI (Pearson correlation coefficient *r* = −0.416, *p* = 0.006) or PAR (Pearson correlation coefficient *r* = −0.355, *p* = 0.021) (no correlation for FLX; Figure 9D–9F). Therapeutic outcome also significantly correlated with the induction of LC3B-II/I in the case of treatment with PAR (Pearson correlation coefficient *r* = 0.453, *p* = 0.020), but not in the case of treatment with AMI or FLX (Figure 9G–9I). Thus, the levels of FKBP51 and Beclin1 at admission, as well as the response to antidepressant treatment in PBMCs, correlate with therapeutic outcome.

## Discussion

In this study, we revealed a novel function of FKBP51 in directing autophagic processes. Further, FKBP51 is required for the effects of both acute and chronic antidepressant treatment on behavior and on autophagic pathways in mice, and for the effects of antidepressants on autophagy in cells. In humans, treatment response in patients could be predicted by the expression of FKBP51, phosphorylated Akt, and Beclin1 in lymphocytes, and in particular by the response of autophagic markers to antidepressant treatment of lymphocytes cultivated ex vivo.

**Figure 10:**
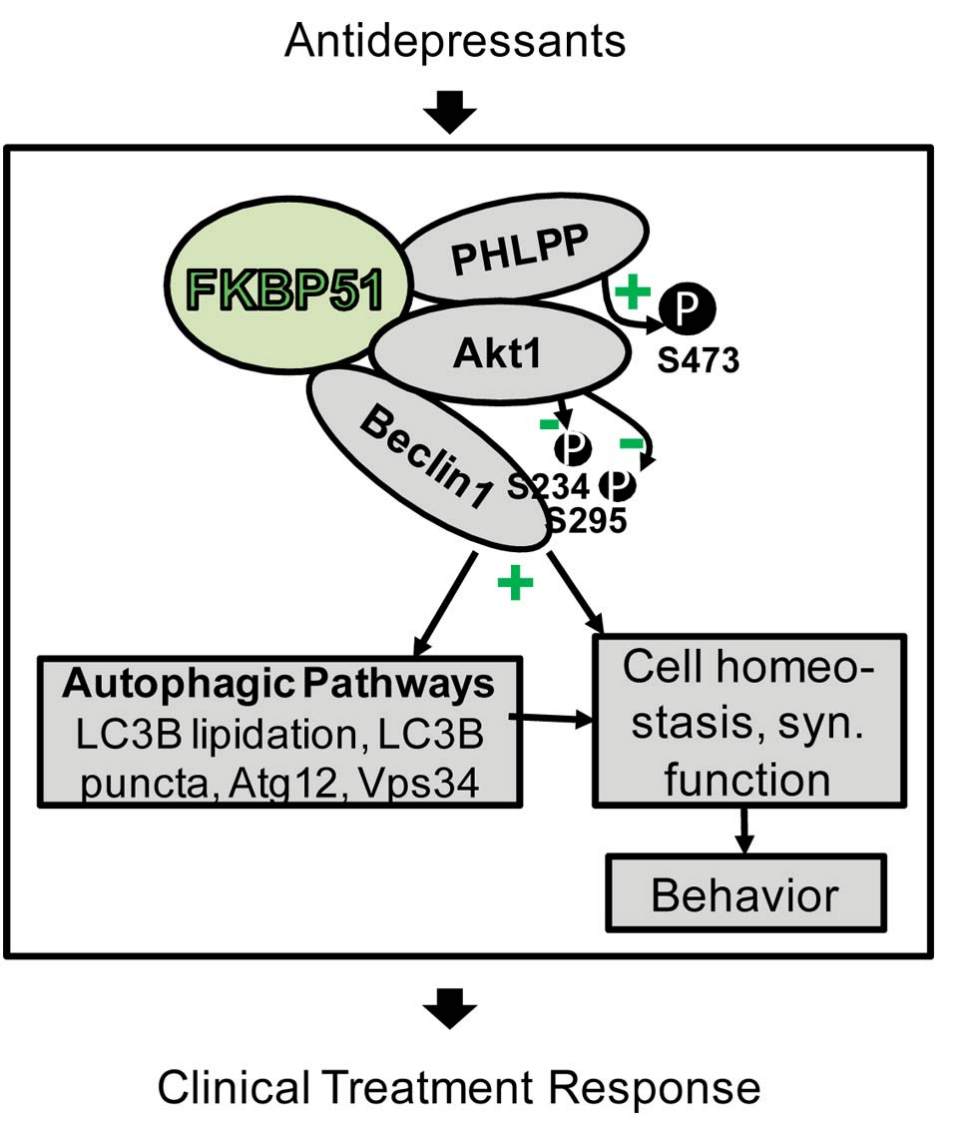
Model of FKBP51's impact on Beclin1 and autophagy pathways. FKBP51 interacts with PHLPP, Akt, and Beclin1. Since PHLPP dephosphorylates Akt (S473 in Akt1), inactive Akt is recruited to Beclin1. This results in lower phosphorylation of Beclin1 and, thus, the induction of autophagic pathways [19]. Autophagic pathways, and thereby also FKBP51, are linked to cell homeostasis and to synaptic (syn.) function [66] as a physiological correlate of behavior [71]. Antidepressants act on the same pathways in an FKBP51-dependent manner and change FKBP51 protein interactions; this could form the basis for the FKBP51 dependency of antidepressant effects in cells, animals, and humans.

FKBP51 was originally selected as a candidate for genetic analyses in depression and antidepressant responsiveness [8] because it is involved in the regulation of the glucocorticoid receptor [7], and thereby the stress hormone axis [61]. Our study brings novel FKBP51-directed pathways to the fore (Figure 10), and establishes the importance of FKBP51 in antidepressant action at the molecular, cellular, and organismic level. It also identifies a potential molecular mechanism for the previously suggested [8] positive correlation between FKBP51 expression and antidepressant treatment response.

The impact of FKBP51 on autophagy established in this study significantly expands the range of actions of this versatile protein to be considered relevant for antidepressant action. Our protein interaction analyses support the model (Figure 10) that FKBP51, as a scaffolding protein, recruits Akt to Beclin1; since FKBP51 also binds to PHLPP [53], inactive Akt is recruited to Beclin1. This results in decreased phosphorylation of Beclin1, thereby shifting Beclin1's action to trigger autophagy [19]. FKBP51-mediated increase in overall Beclin1 levels constitutes a further route by which FKBP51 facilitates autophagy.

The regulation of FKBP51 by glucocorticoids [3],[59] potentially links all the molecular actions of FKBP51 to stress. Our data provide strong evidence that up-regulation of FKBP51 mediates DEX-induced autophagy and dephosphorylation of pAkt^S473^ [60]. Akt, in turn, has been reported previously as a target of antidepressant action, and both positive and negative changes in pAkt^S473^ levels have been found for different antidepressants in various cellular systems [55],[56],[62],[63]. Treatment duration and other factors may account for the divergent results. Based on our findings, we propose that FKBP51 expression status determines whether and how antidepressants alter pAkt^S473^. In general, pAkt^S473^ is under multifactorial control [64] that could be the basis for both inhibitory and FKBP51-dependent stimulatory actions of antidepressants.

In light of the still incompletely understood molecular mechanism by which antidepressants act, our results highlight an FKBP51-dependent intracellular molecular mechanism of antidepressant effects that might complement the known actions on monoaminergic neurotransmitter systems [65]. Nevertheless, given the complexity of the disease and the plethora of reported molecular effects of antidepressants, it is unlikely that all antidepressants, current or future, will depend on FKBP51 or any other single protein. Similarly, some antidepressants may not engage the autophagic pathway, such as the newly suggested fast-acting antidepressants ketamine and scopolamine [57],[58], which did not increase Beclin1 levels.

Autophagy is considered a fundamental process ensuring the functional integrity and fitness of cells and tissues [17]. Recently, a link has been discovered from autophagy to neurotransmission [66], which likely modulates neuronal network dynamics and, ultimately, behavior [67]. Thus, it is possible that both neurotransmission and general cell homeostasis are ramifications of FKBP51-governed autophagy pathways underlying the here described FKBP51 dependency of PAR's effect on body weight gain, stress-coping, and social behavior, and the FKBP51-dependency of clinical antidepressant response.

Consistent with previous reports [5],[6], we found no changes in depression-like behavior in FKBP51-deleted (51KO) mice under basal conditions. In partial contrast to these results in young mice (3–5 mo of age), older 51KO mice (17–20 mo of age) exhibited shorter immobility times in the behavioral despair tests forced swimming and tail suspension [68]. Whether this reflects an age-dependent adaptation of the animals to FKBP51 deletion or whether FKBP51 may exert age-dependent effects will be interesting to investigate. It is worth noting that FKBP51 expression has been shown to increase with age [69].

One of the limitations of the study is the use of only men for the association study of FKBP51 and markers of autophagy and of FKBP51-dependent antidepressant effects in PBMCs. However, the pathway connections and FKBP51 dependency were confirmed in the cohort of patients with depression that comprised women and men. Similarly, only male mice were analyzed in the animal behavior experiments. Further, it should be noted that the clinical sample used for the protein analyses is small (*n* = 51). In addition, there is a general consensus that animal models for depression or antidepressant action do not fully replicate the situation in humans, but only model certain endophenotypes. Finally, it will be interesting to test in future studies whether the use of other autophagy-triggering substances elicits antidepressant-like effects, preferably substances that increase the levels of Beclin1.

While genetic studies of complex diseases, including studies of respective drug actions, have significantly benefitted from the advanced technologies of genome-wide genotyping, the necessity of complementary approaches, such as molecular pathways and network analyses, to move from genomic localization to mechanistic insight has come into focus only recently [70]. Overall, our finding of FKBP51-dependent antidepressants' effects on intracellular pathways, brain function, and behavior strengthens the relevance of the genetic association of FKBP51 with antidepressant response and, furthermore, substantiates the need for more targeted genetic studies. In addition, our results suggest considering autophagy-initiating mechanisms as a pharmacological target to improve the treatment of depression, as has already been discussed for other diseases [20]. Finally, our data call for replication in an independent sample to validate the usefulness of the protein expression status of autophagic factors—as well as the response of these factors to antidepressant treatment of ex vivo cultivated lymphocytes—as novel biomarkers predicting antidepressant efficacy in patients with depression.

## Supporting Information

Figure S1 **Representative Western blots for** **Figure 1C–1G** **(A) and** **Figure 1H–1K** **(B).**
(TIF)

Figure S2 **Functional interaction of FKBP51 with Beclin1, Akt, and PHLPP.** HEK cells were transiently transfected with vector control or a plasmid expressing FKBP51. (A) Numbers indicate the effect of FKBP51 expression on the levels of Beclin1 and on the phosphorylation status of Akt^S473^ and Akt^T308^, corresponding to Figure 2B. Three independent experiments were performed. (B) Representative Western blots of Beclin1 immunoprecipitations from transfected cells and analysis of pBeclin1^S234,S295^ and co-precipitating pAkt^S473^/Akt are displayed (corresponding to Figure 2C). The antibodies directed against phosphorylated Beclin1 worked only after immunoprecipitation [19]. The table summarizes the results of three independent CoIPs and Western blot detections. In the case of Akt and pAkt^S473^, numbers indicate relative intensities of interaction with Beclin1 (intensity of vector control was set to 1), including *p*-values of interaction differences. In the case of pBeclin1^S234,S295^, numbers indicate the fold change in the presence or absence of ectopic FKBP51 (vector control set to 1), including *p*-values.
(TIF)

Figure S3 **Effect of FKBP51 on autophagic flux.** (A) Titration of bafilomycin A_1_. Rat cortical astrocytes were incubated with increasing concentrations of bafilomycin A_1_ as indicated for 2 h. Levels of LC3B-I, LC3B-II, and Actin were determined in protein extracts by Western blotting. (B) FKBP51 enhances autophagic flux. Rat cortical astrocytes were transfected with FKBP51 or vector control and treated with bafilomycin A_1_ as indicated; the levels of LC3B-II/Actin were determined, yielding results similar to the LC3B-II/I ratio (Figure 2F). Representative Western blot corresponding to Figure 2F also presented. *p<0.05. See Table S1 for all statistical parameters.
(TIF)

Figure S4 **Concentrations of amitriptyline and paroxetine needed to elicit markers of autophagy.** Primary cortical astrocytes were treated with PAR (A) or AMI (B) at the concentrations indicated for 72 h. Protein levels were determined for pAkt^S473^/Akt, Beclin1, Atg12, and LC3B-II/I. Representative Western blots are shown. Graphs display the relative abundance measured in three independent experiments. *p<0.05; **p<0.01; ***p<0.001.
(TIF)

Figure S5 **Convergent effects of FKBP51 and antidepressants on protein interactions and pAkt^S473^ in cells.** (A) The interaction of FKBP51 with Beclin1 and Akt was analyzed by Western blotting after CoIP from HEK cells cultivated in the presence or absence of AMI or FLX (10 µM each) for 72 h. Table indicates changes in protein interaction relative to vehicle treatment and includes also PAR (corresponding to Figure 3A). (B and C) Cortical astrocytes were transfected with an FKBP51 expression or control plasmid and treated with PAR, AMI, or FLX (10 µM each) for 72 h; levels of pAkt^S473^/Akt were determined by Western blotting (representative blot in [B], also corresponding to Figure 3B). Protein levels in control-transfected untreated cells were set to 1 (dashed line). (D–F) Wild-type MEFs, 51KO MEFs, and 51KO MEFs transfected with an FKBP51-expressing plasmid were treated AMI, FLX, or PAR (10 µM each) for 72 h, and the pAkt^S473^/Akt and LC3B-II/I ratios were determined by Western blot (representative blot in [D], corresponding to Figure 3C and 3D). Protein levels in wild-type untreated cells were set to 1 (dashed line). *p<0.05. See Table S1 for all statistical parameters.
(TIF)

Figure S6 **Convergent effects of FKBP51 and antidepressants on LC3B-II/I, Beclin1, and Atg12 in cells.** (A and B) Wild-type MEFs, Akt1/2KO MEFs, and Akt1/2KO MEFs transfected with Akt1- and Akt2-expressing plasmids were treated with AMI, FLX, or PAR (10 µM each) for 72 h. The ratio of LC3B-II/I was determined by Western blot (representative example in [B]). LC3B-II/I in the respective untreated cells was set to 1 (dashed line). (C and D) primary rat cortical astrocytes were transfected with a vector expressing GFP-LC3B, in combination with an FKBP51-expressing vector or cloning vector, and treated with AMI or FLX (10 µM each) for 72 h. The number of GFP-LC3B-positive puncta was determined per cell (C). 15–25 randomly selected cells were evaluated for each condition (representative fluorescence images in [D]). (E) Cortical astrocytes were transfected with an FKBP51 expression or control plasmid, and treated with AMI, FLX, or PAR (10 µM each) for 72 h; the levels of Beclin1, LC3B-II/I, and Atg12 were determined by Western blotting (PAR data displayed in Figure 3G–3I; representative blot in Figure S5B). Protein levels in vector-transfected untreated cells were set to 1 (dashed lines). *p<0.05; **p<0.01; ***p<0.001. See Table S1 for all statistical parameters.
(TIF)

Figure S7 **Antidepressants elicit autophagic markers after 45 min in primary astrocytes.** Primary cortical astrocytes were incubated for 45 min with vehicle or with 10 µM of the antidepressant indicated. Protein levels of the indicated autophagy marker proteins were determined by Western blotting. Representative Western blot is provided. Protein levels in untreated cells were set to 1 (dashed lines). *p<0.05; **p<0.01; ***p<0.001. See Table S1 for all statistical parameters.
(TIF)

Figure S8 **Antidepressants enhance autophagic flux.** Rat cortical astrocytes were incubated with the drugs as indicated for 2 h, and the levels of LC3B-I, LC3B-II, and Actin were determined in protein extracts by Western blotting. The graphs display the ratios of LC3B-II/I and of LC3B-II/Actin from three independent experiments. The LC3B-II/I ratio corresponding to the LC3B-II/Actin ratio of (C) is displayed in Figure 3J. The levels in the presence of bafilomycin A_1_ were set to 1 (dashed lines). *p<0.05; **p<0.01; ***p<0.001. See Table S1 for all statistical parameters. BafA1, bafilomycin A_1_; RAP, rapamycin.
(TIF)

Figure S9 **Paroxetine and FKBP51 enhance Beclin1 in primary neuronal cells.** Primary cortical neurons were transfected with vector or FKBP51-expressing plasmid and treated with PAR (10 µM) or vehicle for 72 h as indicated. The levels of Beclin1 and Actin were determined by Western blotting. *p<0.05; **p<0.01. See Table S1 for all statistical parameters.
(TIF)

Figure S10 **Autophagic markers are largely unaffected by haloperidol, ketamine, and scopolamine.** (A) C57BL/6 mice (*n* = 6) were intraperitoneally injected with haloperidol (0.2 mg/kg) or saline and sacrificed 45 min later for preparation of brain extracts. (B) Rat cortical astrocytes were treated with ketamine (30 µM or 100 µM, left), scopolamine (1 µM or 3 µM, right), or vehicle for 45 min, and protein extracts were prepared. For (A and B), protein levels of autophagy markers were determined by Western blotting (representative blots are provided). *p<0.05; **p<0.01. See Table S1 for all statistical parameters. Ket, ketamine; SCO, scopolamine.
(TIF)

Figure S11 **FKBP51 shapes the neuroendocrine effects of chronic stress and chronic paroxetine treatment.** Time course is provided in Figure 4A. When the three-way ANOVA indicated an interaction effect (*p*<0.1), genotype-dependent stress and treatment effects were isolated by normalizing the data to either unstressed controls or vehicle treatment. (A) CSDS resulted in significantly increased basal corticosterone levels in wild-type mice, but not in 51KO mice (*p*<0.05). (B) Basal corticosterone levels assessed 3 wk after the stressor were significantly lower in 51KO mice. (C) Basal corticosterone levels of 51KO mice were less affected by the long-lasting effects of CSDS. (D) PAR decreased basal corticosterone, especially in 51KO mice. (E and F) Circulating corticosterone was significantly decreased in 51KO mice in response to an acute stressor, as well as after a 90-min recovery period Asterisks indicate significant result for planned contrast test for main genotype effect (#), main treatment effect ($), or main condition effect (+): *p<0.05. See Table S1 for all statistical parameters.
(TIF)

Figure S12 **FKBP51 dependendency of autophagy pathway components in human PBMCs and of the effects of dexamethasone and antidepressants in primary astrocytes and in human PBMCs.** (A) Protein extracts from PBMCs of healthy individuals were analyzed for expression of FKBP51 and components of the autophagy pathway. Representative Western blot corresponding to Figure 6 is shown. (B) PBMCs were collected from healthy individuals (*n* = 14) before and 6 h after the intake of 1.5 mg of DEX, and proteins were analyzed. Representative Western blot corresponding to Figure 7A and 7B is shown. (C–F) Primary astrocytes from FKBP51^+/+^ and 51KO mice were treated with vehicle or DEX (10 µM for 6 h), and protein levels were determined by Western blot (representative blot in [F]). (G) Representative Western blot of extracts from antidepressant-treated PBMCs (corresponding to Figure 7C–7F and table in [H]). (H) Summary of the correlations of the effects of antidepressants on autophagic markers with the expression of FKBP51 in PBMCs from healthy individuals.
(TIF)

Table S1 **Details of the results of the ANOVA analyses.**
(PDF)

Table S2 **Details of the results of the regression analyses.**
(PDF)

Text S1 **STROBE statement.**
(DOCX)

Text S2 **ARRIVE guidelines checklist.**
(DOC)

Text S3 **Extended information on Western blot quantification.**
(DOCX)

## Abbreviations

51KO: FKBP51 knockout
Akt1/2KO: Akt1/2 knockout
AMI: amitriptyline
ANOVA: analysis of variance
CoIP: co-immunoprecipitation
CSDS: chronic social defeat stress
DEX: dexamethasone
FLX: fluoxetine
FST: forced swim test
HDRS: Hamilton Depression Rating Scale
MARS: Munich Antidepressant Response Signature
MEF: mouse embryonic fibroblast
PAR: paroxetine
PBMC: peripheral blood mononuclear cell
SD: standard deviation
