# Supplementary figures and images for: Association of FKBP51 with Priming of Autophagy Pathways and Mediation of Antidepressant Treatment Response: Evidence in Cells, Mice, and Humans
Source: PLoS Med. 2014 Nov 11;11(11):e1001755. doi: 10.1371/journal.pmed.1001755 (PMC4227651; doi:10.1371/journal.pmed.1001755)

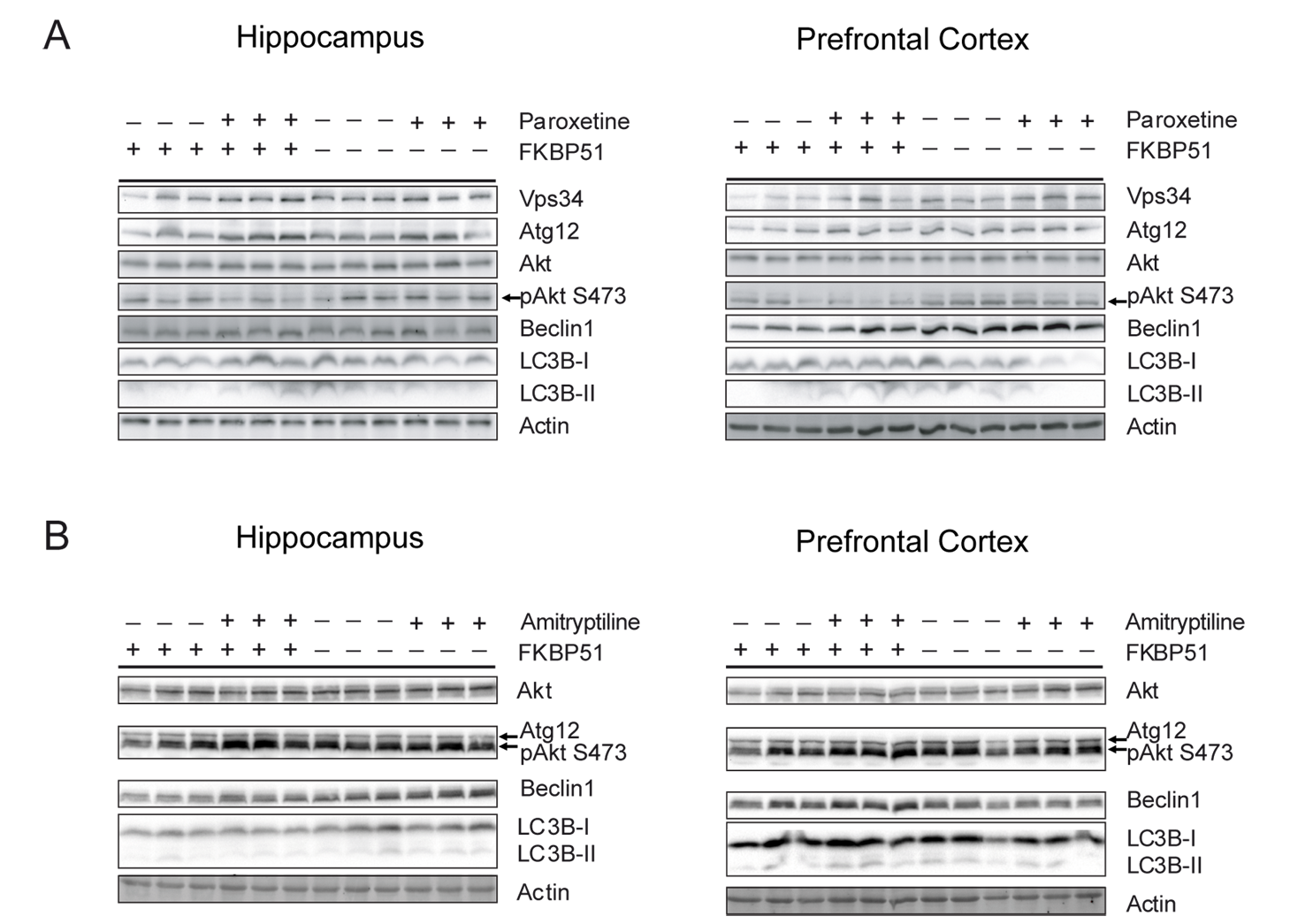

Supplement: Figure S1 — Representative Western blots for Figure 1C–1G (A) and Figure 1H–1K (B). (TIF) [file pmed.1001755.s001.tif]

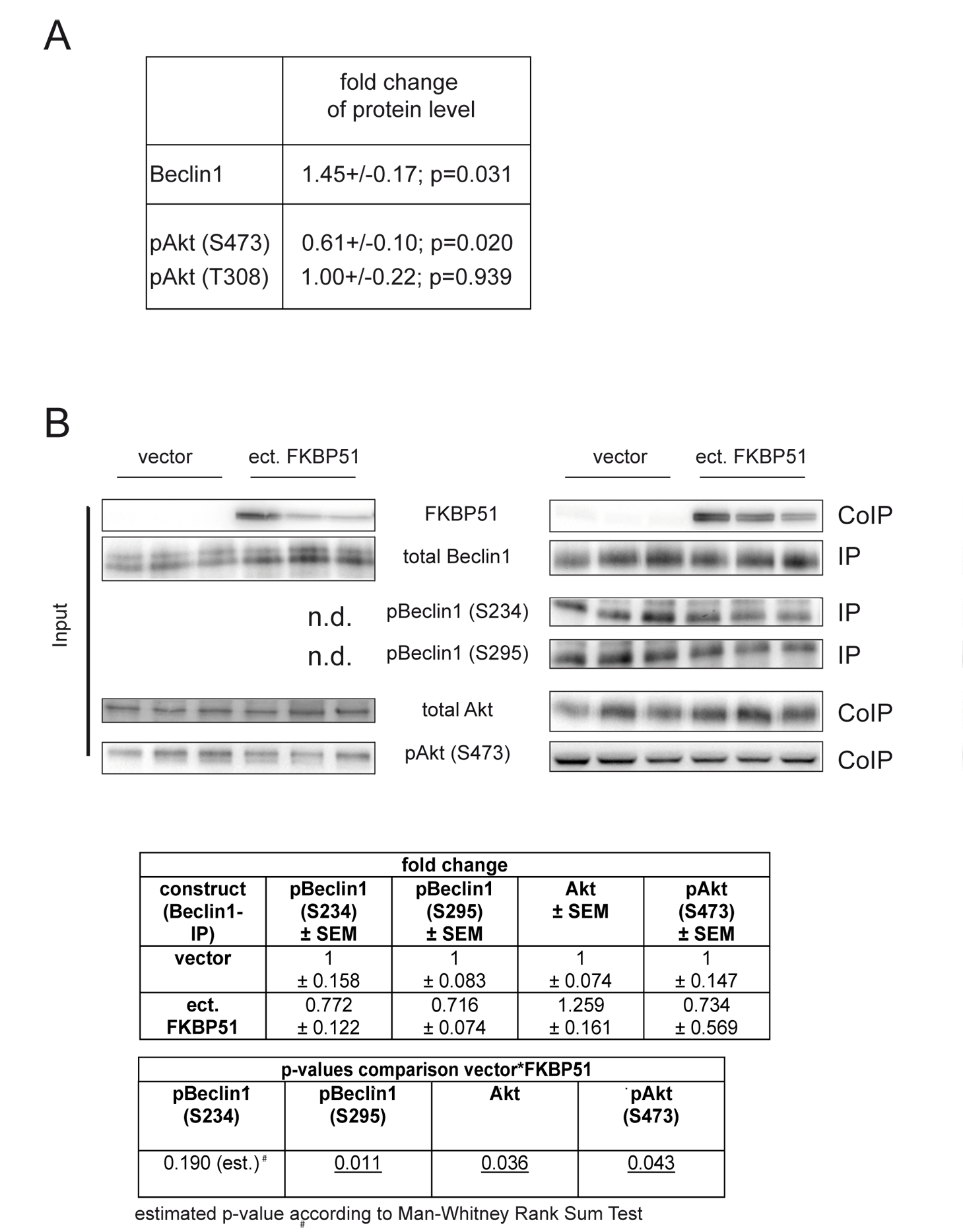

Supplement: Figure S2 — Functional interaction of FKBP51 with Beclin1, Akt, and PHLPP. HEK cells were transiently transfected with vector control or a plasmid expressing FKBP51. (A) Numbers indicate the effect of FKBP51 expression on the levels of Beclin1 and on the phosphorylation status of AktS473 and AktT308, corresponding to Figure 2B. Three independent experiments were performed. (B) Representative Western blots of Beclin1 immunoprecipitations from transfected cells and analysis of pBeclin1S234,S295 and co-precipitating pAktS473/Akt are displayed (corresponding to Figure 2C). The antibodies directed against phosphorylated Beclin1 worked only after immunoprecipitation [19]. The table summarizes the results of three independent CoIPs and Western blot detections. In the case of Akt and pAktS473, numbers indicate relative intensities of interaction with Beclin1 (intensity of vector control was set to 1), including p-values of interaction differences. In the case of pBeclin1S234,S295, numbers indicate the fold change in the presence or absence of ectopic FKBP51 (vector control set to 1), including p-values. (TIF) [file pmed.1001755.s002.tif]

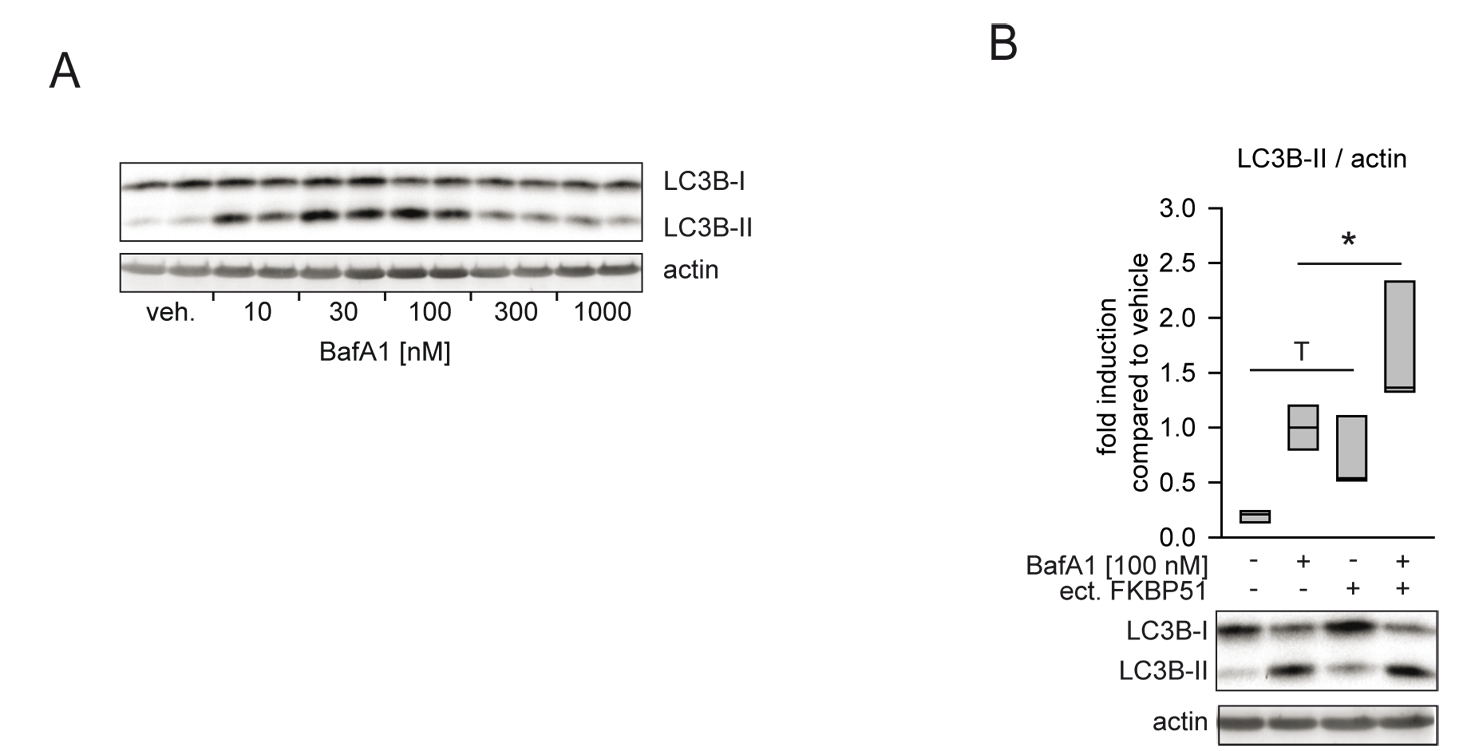

Supplement: Figure S3 — Effect of FKBP51 on autophagic flux. (A) Titration of bafilomycin A1. Rat cortical astrocytes were incubated with increasing concentrations of bafilomycin A1 as indicated for 2 h. Levels of LC3B-I, LC3B-II, and Actin were determined in protein extracts by Western blotting. (B) FKBP51 enhances autophagic flux. Rat cortical astrocytes were transfected with FKBP51 or vector control and treated with bafilomycin A1 as indicated; the levels of LC3B-II/Actin were determined, yielding results similar to the LC3B-II/I ratio (Figure 2F). Representative Western blot corresponding to Figure 2F also presented. *p<0.05. See Table S1 for all statistical parameters. (TIF) [file pmed.1001755.s003.tif]

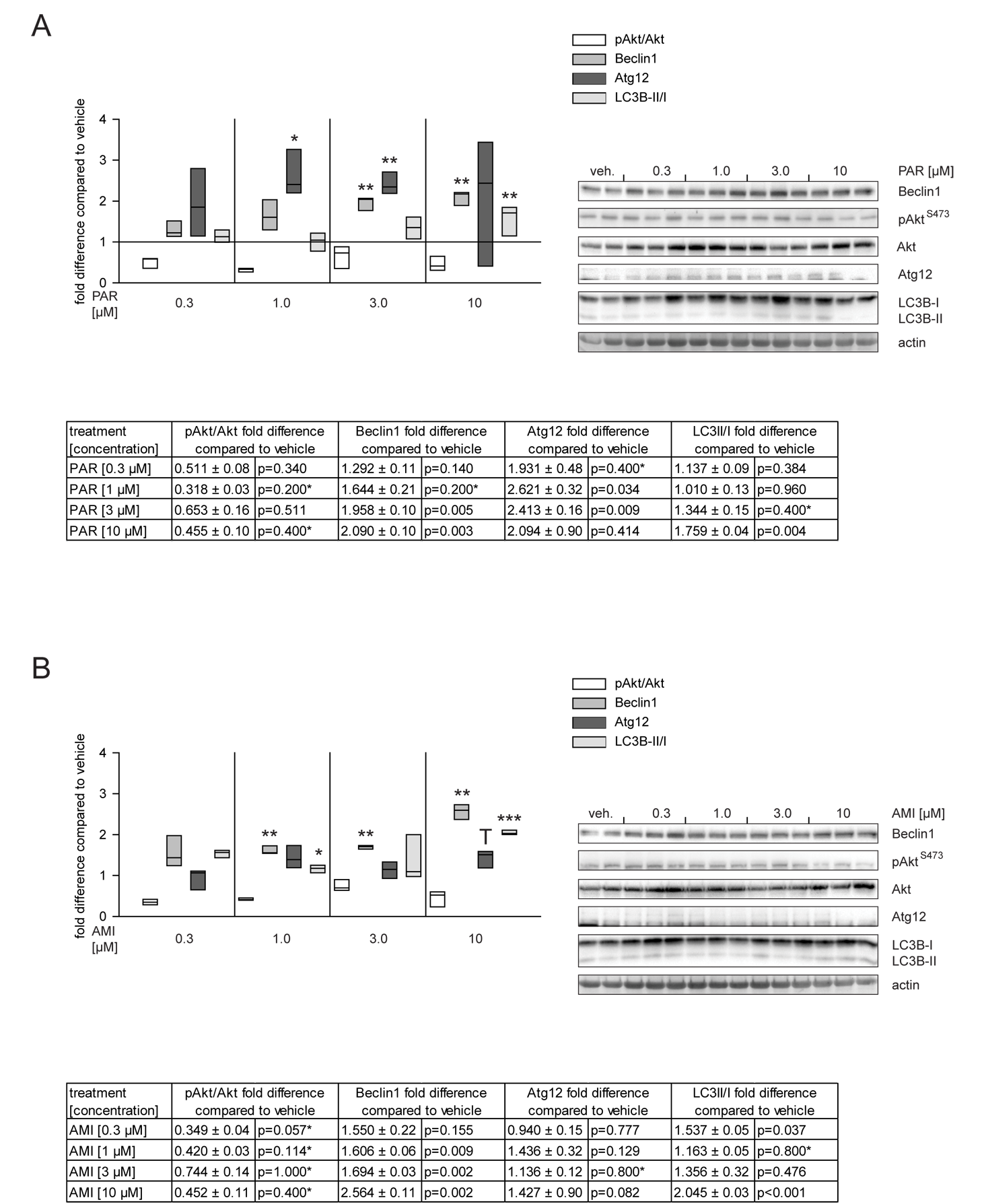

Supplement: Figure S4 — Concentrations of amitriptyline and paroxetine needed to elicit markers of autophagy. Primary cortical astrocytes were treated with PAR (A) or AMI (B) at the concentrations indicated for 72 h. Protein levels were determined for pAktS473/Akt, Beclin1, Atg12, and LC3B-II/I. Representative Western blots are shown. Graphs display the relative abundance measured in three independent experiments. *p<0.05; **p<0.01; ***p<0.001. (TIF) [file pmed.1001755.s004.tif]

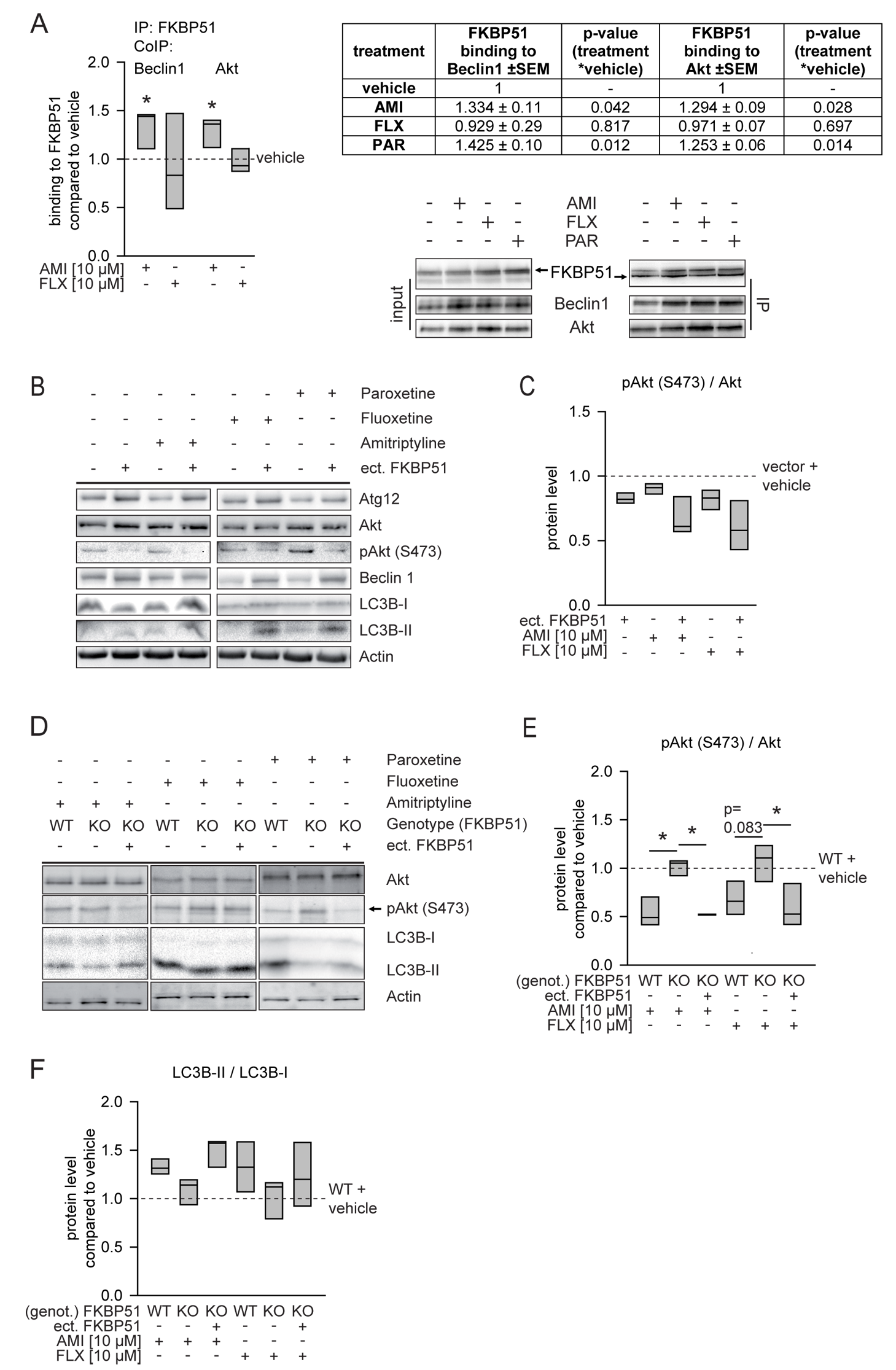

Supplement: Figure S5 — Convergent effects of FKBP51 and antidepressants on protein interactions and pAktS473 in cells. (A) The interaction of FKBP51 with Beclin1 and Akt was analyzed by Western blotting after CoIP from HEK cells cultivated in the presence or absence of AMI or FLX (10 µM each) for 72 h. Table indicates changes in protein interaction relative to vehicle treatment and includes also PAR (corresponding to Figure 3A). (B and C) Cortical astrocytes were transfected with an FKBP51 expression or control plasmid and treated with PAR, AMI, or FLX (10 µM each) for 72 h; levels of pAktS473/Akt were determined by Western blotting (representative blot in [B], also corresponding to Figure 3B). Protein levels in control-transfected untreated cells were set to 1 (dashed line). (D–F) Wild-type MEFs, 51KO MEFs, and 51KO MEFs transfected with an FKBP51-expressing plasmid were treated AMI, FLX, or PAR (10 µM each) for 72 h, and the pAktS473/Akt and LC3B-II/I ratios were determined by Western blot (representative blot in [D], corresponding to Figure 3C and 3D). Protein levels in wild-type untreated cells were set to 1 (dashed line). *p<0.05. See Table S1 for all statistical parameters. (TIF) [file pmed.1001755.s005.tif]

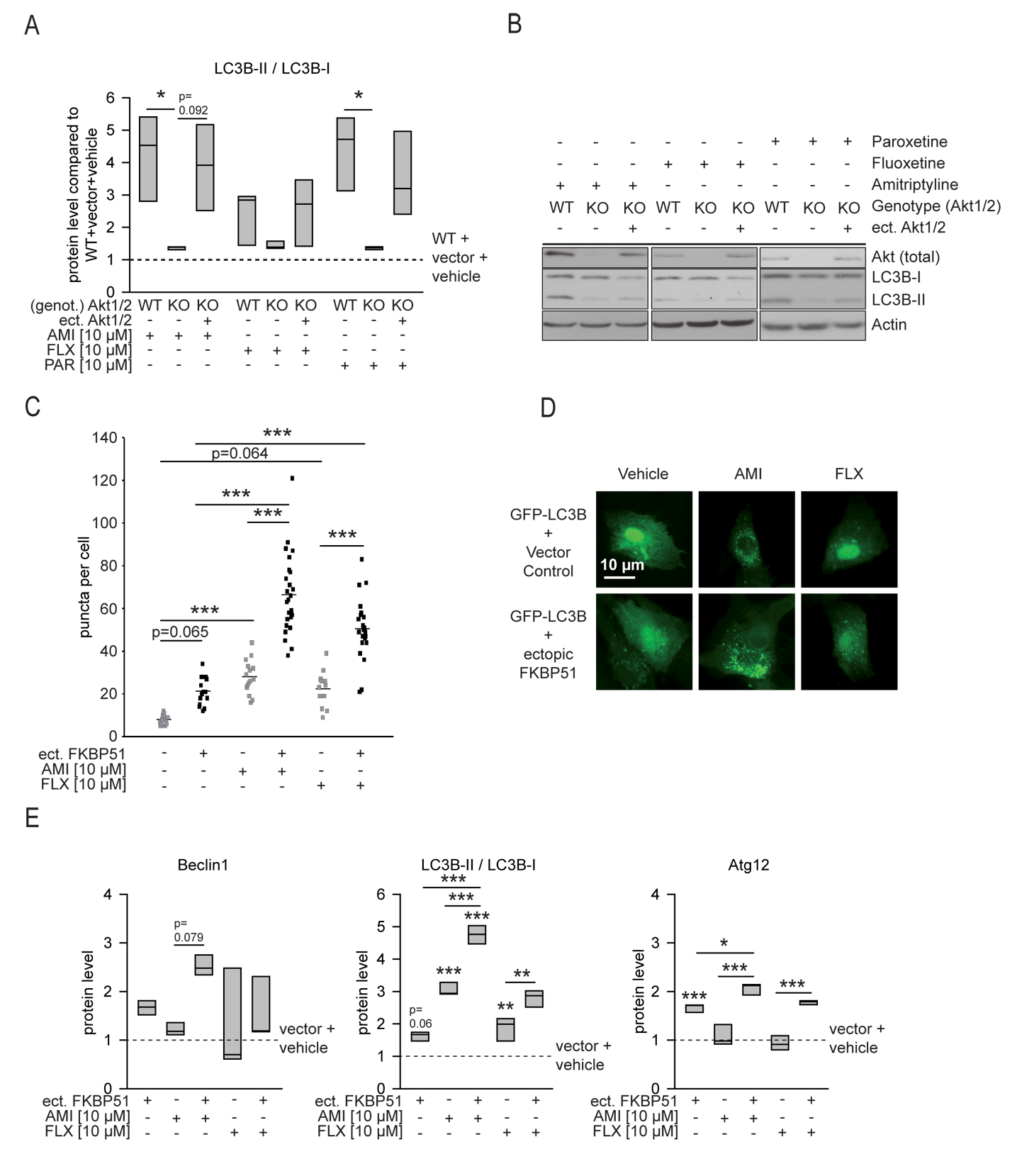

Supplement: Figure S6 — Convergent effects of FKBP51 and antidepressants on LC3B-II/I, Beclin1, and Atg12 in cells. (A and B) Wild-type MEFs, Akt1/2KO MEFs, and Akt1/2KO MEFs transfected with Akt1- and Akt2-expressing plasmids were treated with AMI, FLX, or PAR (10 µM each) for 72 h. The ratio of LC3B-II/I was determined by Western blot (representative example in [B]). LC3B-II/I in the respective untreated cells was set to 1 (dashed line). (C and D) primary rat cortical astrocytes were transfected with a vector expressing GFP-LC3B, in combination with an FKBP51-expressing vector or cloning vector, and treated with AMI or FLX (10 µM each) for 72 h. The number of GFP-LC3B-positive puncta was determined per cell (C). 15–25 randomly selected cells were evaluated for each condition (representative fluorescence images in [D]). (E) Cortical astrocytes were transfected with an FKBP51 expression or control plasmid, and treated with AMI, FLX, or PAR (10 µM each) for 72 h; the levels of Beclin1, LC3B-II/I, and Atg12 were determined by Western blotting (PAR data displayed in Figure 3G–3I; representative blot in Figure S5B). Protein levels in vector-transfected untreated cells were set to 1 (dashed lines). *p<0.05; **p<0.01; ***p<0.001. See Table S1 for all statistical parameters. (TIF) [file pmed.1001755.s006.tif]

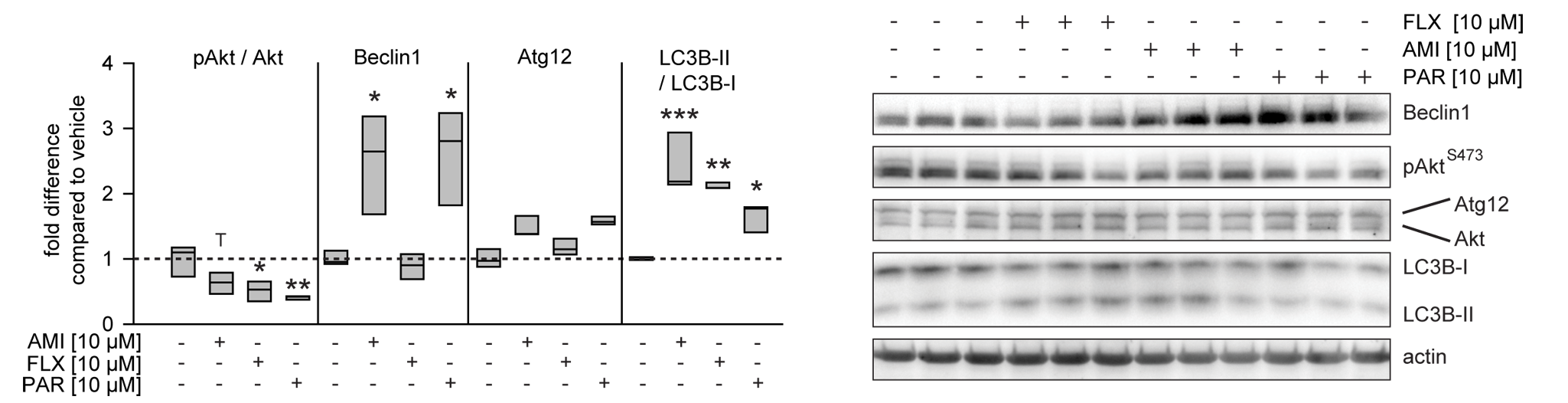

Supplement: Figure S7 — Antidepressants elicit autophagic markers after 45 min in primary astrocytes. Primary cortical astrocytes were incubated for 45 min with vehicle or with 10 µM of the antidepressant indicated. Protein levels of the indicated autophagy marker proteins were determined by Western blotting. Representative Western blot is provided. Protein levels in untreated cells were set to 1 (dashed lines). *p<0.05; **p<0.01; ***p<0.001. See Table S1 for all statistical parameters. (TIF) [file pmed.1001755.s007.tif]

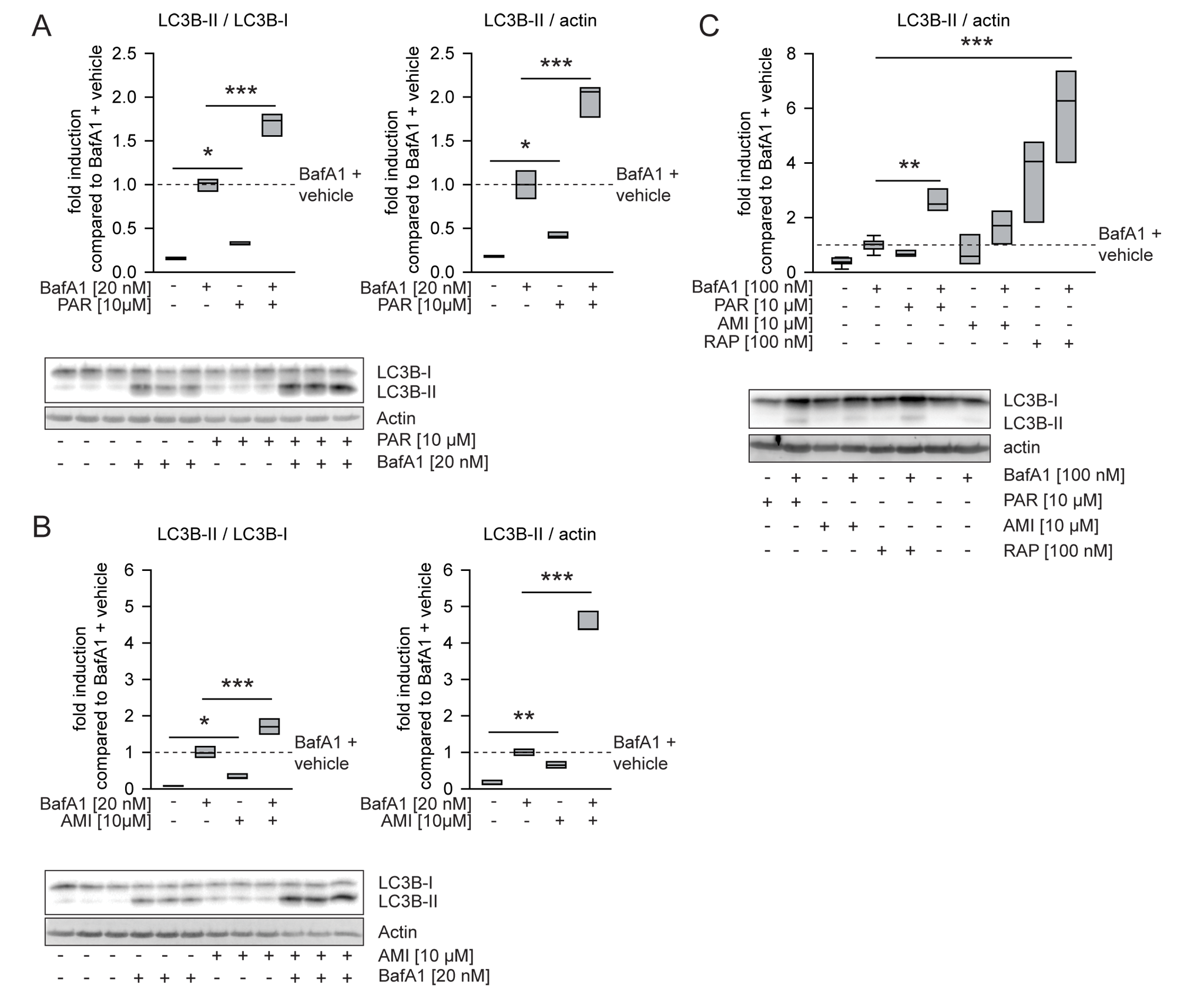

Supplement: Figure S8 — Antidepressants enhance autophagic flux. Rat cortical astrocytes were incubated with the drugs as indicated for 2 h, and the levels of LC3B-I, LC3B-II, and Actin were determined in protein extracts by Western blotting. The graphs display the ratios of LC3B-II/I and of LC3B-II/Actin from three independent experiments. The LC3B-II/I ratio corresponding to the LC3B-II/Actin ratio of (C) is displayed in Figure 3J. The levels in the presence of bafilomycin A1 were set to 1 (dashed lines). *p<0.05; **p<0.01; ***p<0.001. See Table S1 for all statistical parameters. BafA1, bafilomycin A1; RAP, rapamycin. (TIF) [file pmed.1001755.s008.tif]

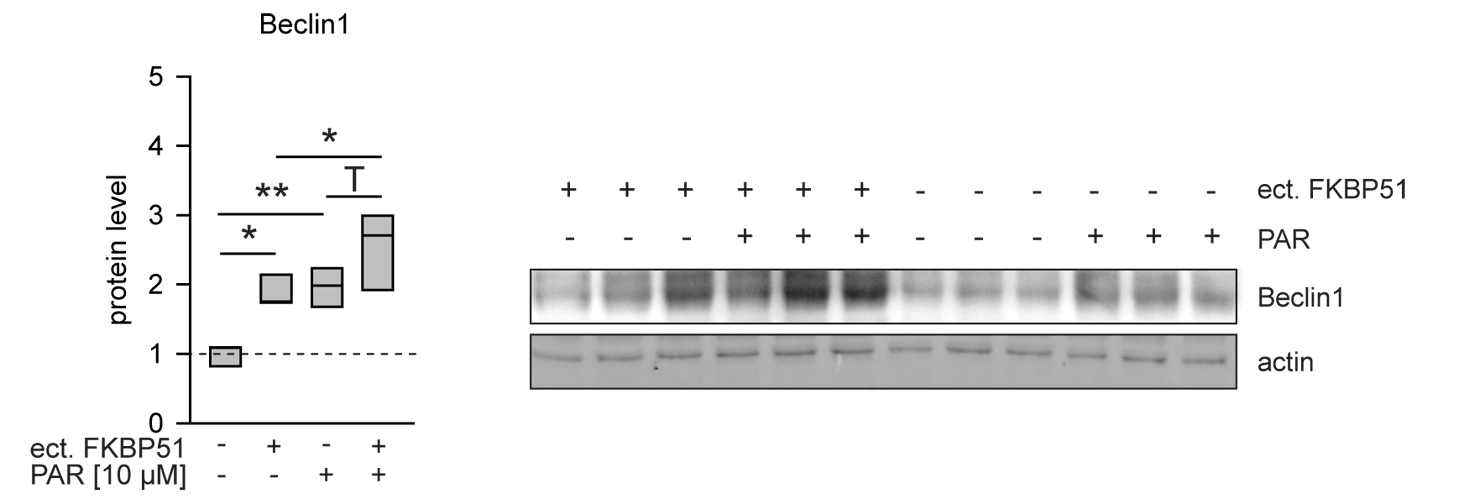

Supplement: Figure S9 — Paroxetine and FKBP51 enhance Beclin1 in primary neuronal cells. Primary cortical neurons were transfected with vector or FKBP51-expressing plasmid and treated with PAR (10 µM) or vehicle for 72 h as indicated. The levels of Beclin1 and Actin were determined by Western blotting. *p<0.05; **p<0.01. See Table S1 for all statistical parameters. (TIF) [file pmed.1001755.s009.tif]

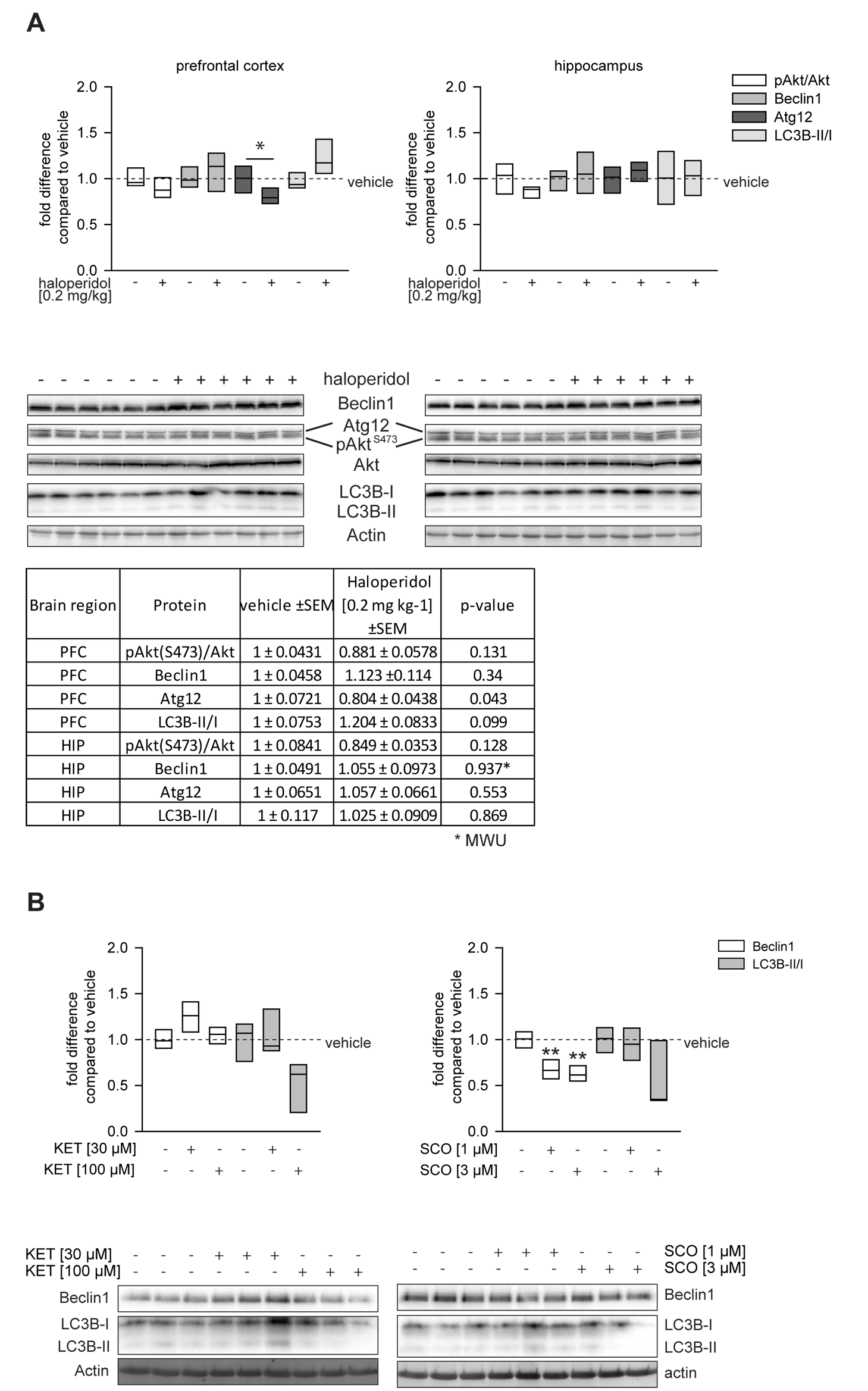

Supplement: Figure S10 — Autophagic markers are largely unaffected by haloperidol, ketamine, and scopolamine. (A) C57BL/6 mice (n = 6) were intraperitoneally injected with haloperidol (0.2 mg/kg) or saline and sacrificed 45 min later for preparation of brain extracts. (B) Rat cortical astrocytes were treated with ketamine (30 µM or 100 µM, left), scopolamine (1 µM or 3 µM, right), or vehicle for 45 min, and protein extracts were prepared. For (A and B), protein levels of autophagy markers were determined by Western blotting (representative blots are provided). *p<0.05; **p<0.01. See Table S1 for all statistical parameters. Ket, ketamine; SCO, scopolamine. (TIF) [file pmed.1001755.s010.tif]

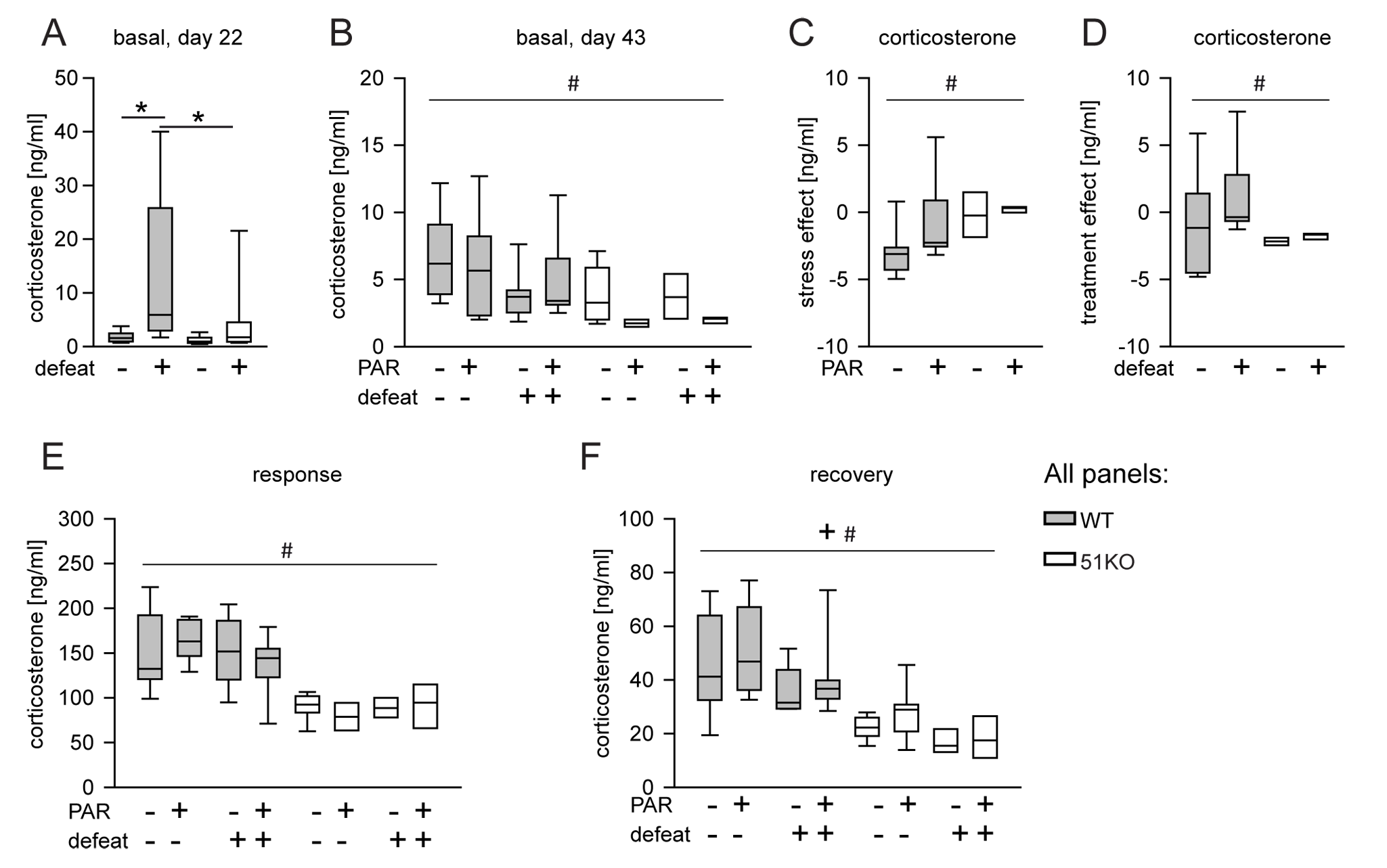

Supplement: Figure S11 — FKBP51 shapes the neuroendocrine effects of chronic stress and chronic paroxetine treatment. Time course is provided in Figure 4A. When the three-way ANOVA indicated an interaction effect (p<0.1), genotype-dependent stress and treatment effects were isolated by normalizing the data to either unstressed controls or vehicle treatment. (A) CSDS resulted in significantly increased basal corticosterone levels in wild-type mice, but not in 51KO mice (p<0.05). (B) Basal corticosterone levels assessed 3 wk after the stressor were significantly lower in 51KO mice. (C) Basal corticosterone levels of 51KO mice were less affected by the long-lasting effects of CSDS. (D) PAR decreased basal corticosterone, especially in 51KO mice. (E and F) Circulating corticosterone was significantly decreased in 51KO mice in response to an acute stressor, as well as after a 90-min recovery period Asterisks indicate significant result for planned contrast test for main genotype effect (#), main treatment effect ($), or main condition effect (+): *p<0.05. See Table S1 for all statistical parameters. (TIF) [file pmed.1001755.s011.tif]

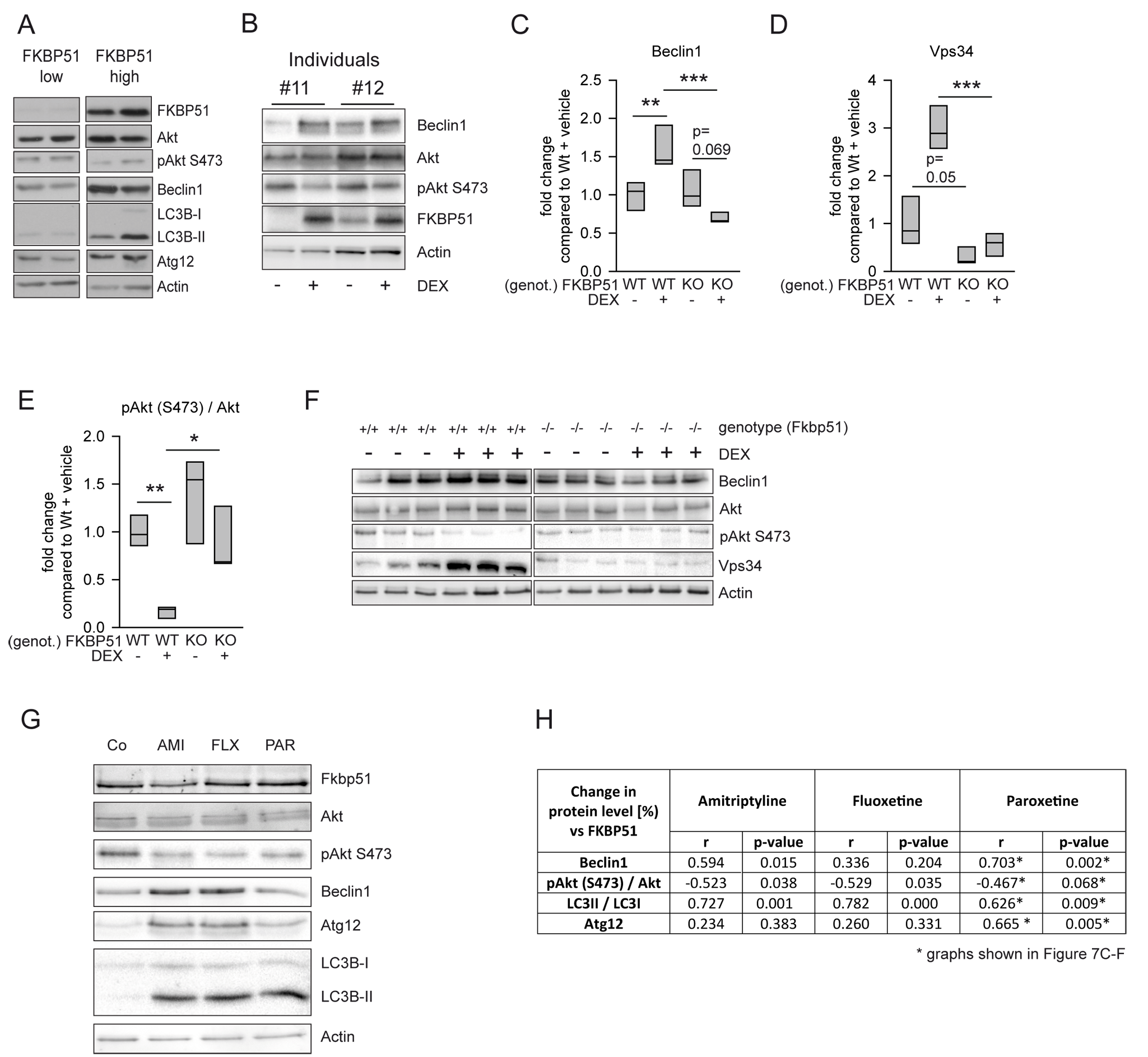

Supplement: Figure S12 — FKBP51 dependendency of autophagy pathway components in human PBMCs and of the effects of dexamethasone and antidepressants in primary astrocytes and in human PBMCs. (A) Protein extracts from PBMCs of healthy individuals were analyzed for expression of FKBP51 and components of the autophagy pathway. Representative Western blot corresponding to Figure 6 is shown. (B) PBMCs were collected from healthy individuals (n = 14) before and 6 h after the intake of 1.5 mg of DEX, and proteins were analyzed. Representative Western blot corresponding to Figure 7A and 7B is shown. (C–F) Primary astrocytes from FKBP51+/+ and 51KO mice were treated with vehicle or DEX (10 µM for 6 h), and protein levels were determined by Western blot (representative blot in [F]). (G) Representative Western blot of extracts from antidepressant-treated PBMCs (corresponding to Figure 7C–7F and table in [H]). (H) Summary of the correlations of the effects of antidepressants on autophagic markers with the expression of FKBP51 in PBMCs from healthy individuals. (TIF) [file pmed.1001755.s012.tif]
