# Supplementary material for: Association of FKBP51 with Priming of Autophagy Pathways and Mediation of Antidepressant Treatment Response: Evidence in Cells, Mice, and Humans
Source: PLoS Med. 2014 Nov 11;11(11):e1001755. doi: 10.1371/journal.pmed.1001755 (PMC4227651; doi:10.1371/journal.pmed.1001755)
Supplement: Table S1 — Details of the results of the ANOVA analyses. (PDF) [file pmed.1001755.s013.pdf]

Table S1, Gassen et al.

Figure	Measure	Test	Independent Factor(s)	Main Effect(s)	F df error	F-value	p-value	PES (partial Eta squared)	Estimated effect size: Cohen's f statistic
1A	Struggling PAR	2-way ANOVA	Genotype, Treatment	Genotype	(1, 27)	13.52	0.001	0.33	0.71
				Treatment		25.752	<0.0001	0.49	0.98
				Geno x Treat		4.247	0.049	0.14	0.40
	Immobility PAR	2-way ANOVA	Genotype, Treatment	Genotype	(1, 27)	10.476	0.003	0.28	0.62
				Treatment		15.234	0.001	0.36	0.75
				Geno x Treat		4.927	0.035	0.15	0.43
1B	Struggling AMI	2-way ANOVA	Genotype, Treatment	Treatment	(1, 34)	7.158	0.011	0.17	0.46
				Geno x Treat		4.654	0.038	0.12	0.37
	Immobility AMI	2-way ANOVA	Genotype, Treatment	Treatment	(1, 34)	15.577	<0.0001	0.36	0.75
1C	Beclin1 HIP PAR	2-way ANOVA	Genotype, Treatment	Treatment	(1, 28)	4.944	0.034	0.15	0.42
				Geno x Treat		4.299	0.047	0.13	0.39
	Beclin1 PFC PAR	2-way ANOVA	Genotype, Treatment	Genotype	(1, 28)	8.827	0.006	0.24	0.56
				Treatment		6.335	0.018	0.19	0.48
				Geno x Treat		25.002	0.0002	0.47	0.95
1D	Atg12 Hip	2-way ANOVA	Genotype, Treatment	Genotype	(1, 28)	12.654	0.001	0.31	0.67
				Treatment		18.858	0.0002	0.40	0.82
				Geno x Treat		29.447	<0.0001	0.51	1.03
	Atg12 PFC	2-way ANOVA	Genotype, Treatment	Genotype	(1, 28)	5.887	0.022	0.17	0.46
				Geno x Treat		9.383	0.005	0.25	0.58
1E	Vps34 Hip	2-way ANOVA	Genotype, Treatment	Genotype	(1, 28)	21.781	<0.0001	0.44	0.88
				Treatment		33.501	<0.0001	0.55	1.09
				Geno x Treat		25.275	<0.0001	0.47	0.95

Figure	Measure	Test	Independent Factor(s)	Main Effect(s)	F df error	F-value	p-value	PES (partial Eta squared)	Estimated effect size: Cohen's f statistic
1E	Vps34 PFC	2-way ANOVA	Genotype, Treatment	Genotype	(1, 28)	11.231	0.002	0.29	0.63
				Treatment		14.334	0.001	0.34	0.72
				Geno x Treat		8.125	0.008	0.23	0.54
1F	LC3B-II / I HIP PAR	2-way ANOVA	Genotype, Treatment	Geno x Treat	(1, 28)	7.967	0.009	0.22	0.53
	LC3B-II / I PFC PAR	2-way ANOVA	Genotype, Treatment	Genotype	(1, 28)	16.671	<0.0001	0.37	0.77
				Treatment		28.243	<0.0001	0.50	1.00
				Geno x Treat		32.117	<0.0001	0.53	1.07
1G	pAkt (S473) / Akt Hip	2-way ANOVA	Genotype, Treatment	Treatment	(1, 28)	4.999	0.034	0.15	0.42
				Geno x Treat		6.508	0.016	0.19	0.48
	pAkt (S473) / Akt PFC	2-way ANOVA	Genotype, Treatment	Genotype	(1, 28)	4.354	0.046	0.14	0.40
				Treatment		17.239	0.0002	0.38	0.78
				Geno x Treat		27.685	<0.0001	0.50	0.99
1H	Beclin1 HIP AMI	2-way ANOVA	Genotype, Treatment	Treatment	(1, 35)	6.593	0.015	0.16	0.43
	Beclin1 PFC AMI	2-way ANOVA	Genotype, Treatment	Genotype	(1, 35)	6.735	0.014	0.16	0.44
				Treatment		10.696	0.002	0.23	0.55
1I	Atg12 Hip	2-way ANOVA	Genotype, Treatment	Treatment	(1, 35)	5.568	0.024	0.14	0.40
				Geno x Treat		5.672	0.023	0.14	0.40
	Atg12 PFC	2-way ANOVA	Genotype, Treatment	Treatment	(1, 35)	4.578	0.039	0.12	0.36
1J	LC3B-II / I HIP AMI	2-way ANOVA	Genotype, Treatment						
	LC3B-II / I PFC AMI	2-way ANOVA	Genotype, Treatment	Geno x Treat	(1, 35)	7.21	0.011	0.17	0.45
1K	pAkt (S473) / Akt Hip	2-way ANOVA	Genotype, Treatment	Genotype	(1, 35)	5.249	0.028	0.13	0.39
	pAkt (S473) / Akt PFC	2-way ANOVA	Genotype, Treatment	Geno x Treat	(1, 35)	4.9	0.033	0.12	0.37

Figure	Measure	Test	Independent Factor(s)	Main Effect(s)	F df error	F-value	p-value	PES (partial Eta squared)	Estimated effect size: Cohen's f statistic
2D	Beclin 1	1-way ANOVA	Genotype	Genotype	(2, 6)	32.996	0.001	0.92	3.32
	LC3B-II / LC3B-I	1-way ANOVA	Genotype	Genotype		177.628	<0.0001	0.98	7.60
	Atg12	1-way ANOVA	Genotype	Genotype		26.092	0.001	0.90	2.95
	pAkt S473	1-way ANOVA	Genotype	Genotype		267.265	<0.0001	0.99	9.48
2F	LC3B-II / LC3B-I	2-way ANOVA	Genotype, Treatment	Genotype	(1, 8)	20.52	0.002	0.72	1.60
				Treatment		86.612	<0.0001	0.92	3.28
2G	Beclin1	2-way ANOVA	FKBP51 Genotype, Akt Genotype	Akt Genotype	(2, 12)	11.729	0.002	0.66	1.40
				FKBP51 Genotype	(1, 12)	24.607	<0.0001	0.67	1.43
				Akt x FKBP51	(2, 12)	19.634	<0.0001	0.77	1.81
	LC3B-II / LC3B-I	2-way ANOVA	FKBP51 Genotype, Akt Genotype	Akt Genotype	(2, 12)	11.107	0.002	0.65	1.36
				FKBP51 Genotype	(1, 12)	105.288	<0.0001	0.90	2.97
				Akt x FKBP51	(2, 12)	13.72	0.001	0.70	1.51
3B	pAkt (S473) / Akt	2-way ANOVA	Genotype, Treatment	Genotype	(1, 8)	203.795	<0.0001	0.96	5.03
				Treatment		126.33	<0.0001	0.94	3.96
				Geno x Treat		12.696	0.007	0.61	1.26
3C	pAkt (S473) / Akt	1-way ANOVA	Genotype	Genotype	(2, 6)	27.712	0.001	0.90	3.03
3D	LC3B-II / LC3B-I	1-way ANOVA	Genotype	Genotype	(2, 6)	12.042	0.008	0.80	2.01
3E	puncta per cell	2-way ANOVA	Genotype, Treatment	Genotype	(1, 67)	106.64	<0.0001	0.61	1.26
				Treatment		173.593	<0.0001	0.72	1.61
				Geno x Treat		29.555	<0.0001	0.31	0.66
3G	Beclin1	2-way ANOVA	Genotype, Treatment	Genotype	(1, 8)	389.829	<0.0001	0.98	7.00
				Treatment		135.849	<0.0001	0.94	4.11
				Geno x Treat		41.673	<0.0001	0.84	2.28

Figure	Measure	Test	Independent Factor(s)	Main Effect(s)	F df error	F-value	p-value	PES (partial Eta squared)	Estimated effect size: Cohen's f statistic
3H	LC3B-II / LC3B-I	2-way ANOVA	Genotype, Treatment	Genotype	(1, 8)	357.568	<0.0001	0.98	6.67
				Treatment		2784.94	<0.0001	1.00	18.23
				Geno x Treat		111.086	<0.0001	0.93	3.73
3I	Atg12	2-way ANOVA	Genotype, Treatment	Genotype	(1, 8)	28.482	0.001	0.78	1.89
				Treatment		15.002	0.005	0.09	0.31
3J	LC3B-II / LC3B-I	2-way ANOVA	Treatment, BafA1	Treatment	(3, 36)	26.795	<0.0001	0.69	1.50
				BafA1	(1, 36)	96.386	<0.0001	0.73	1.64
				Treat x BafA1	(3, 36)	6.554	0.001	0.35	0.74
4B	Body weight day22	2-way ANOVA	Genotype, Condition	Genotype	(1, 81)	17.794	<0.0001	0.18	0.47
				Condition		4.865	0.03	0.06	0.25
4C	Body weight day 43	3-way ANOVA	Genotype, Treatment, Condition	Genotype	(1, 66)	30.177	<0.0001	0.31	0.68
				Treatment		3.752	0.057	0.05	0.24
				Condition		3.857	0.054	0.06	0.24
				Cond x Treat		3.296	0.074	0.05	0.22
4D	Body weight (stress effect)	2-way ANOVA	Genotype, Treatment	Treatment	(1, 33)	5.556	0.024	0.14	0.41
	Body weight (treatment effect)	2-way ANOVA	Genotype, Condition	Genotype	(1, 33)	4.457	0.042	0.12	0.37
				Condition		6	0.02	0.15	0.43
4E	Adrenal weight	3-way ANOVA	Genotype, Treatment, Condition	Genotype	(1, 69)	53.340	<0.0001	0.44	0.88
				Condition		56.652	<0.0001	0.45	0.91
				Geno x Cond		6.245	0.015	0.08	0.30
				Geno x Treat		5.613	0.021	0.08	0.28
4F	Adrenal weight (stress effect)	2-way ANOVA	Genotype, Treatment	Genotype	(1, 35)	12.59	0.001	0.27	0.60
				Treatment		4.41	0.043	0.11	0.36
	Adrenal weight (treatment effect)	2-way ANOVA	Genotype, Treatment	Genotype	(1, 33)	22.181	<0.0001	0.40	0.82
				Condition		8.594	0.006	0.21	0.51
4G	Thymus weight	3-way ANOVA	Genotype, Treatment,	Genotype	(1, 70)	61.684	<0.0001	0.47	0.94
				Condition		9.908	0.002	0.12	0.38

Figure	Measure	Test	Independent Factor(s)	Main Effect(s)	F df error	F-value	p-value	PES (partial Eta squared)	Estimated effect size: Cohen's f statistic
5A	Open field	3-way ANOVA	Genotype, Treatment, Condition						
5B	Social avoidance	3-way ANOVA	Genotype, Treatment, Condition	Condition	(1, 66)	6.084	0.016	0.08	0.30
				Geno x Cond		3.221	0.077	0.05	0.22
				Geno x Treat		4.271	0.043	0.06	0.25
5C	Social avoidance (stress effect)	2-way ANOVA	Genotype, Treatment	Genotype	(1, 33)	10.677	0.003	0.25	0.57
5D	Social avoidance (treatment effect)	2-way ANOVA	Genotype, Condition	Genotype	(1, 33)	5.663	0.024	0.16	0.43
5E	Struggling	3-way ANOVA	Genotype, Treatment, Condition	Genotype	(1, 72)	21.562	<0.0001	0.23	0.55
				Treatment		7.411	0.008	0.09	0.32
				Geno x Treat		3.777	0.056	0.05	0.23
5F	Immobility	3-way ANOVA	Genotype, Treatment, Condition	Geno x Treat	(1, 72)	4.784	0.032	0.06	0.26
5G	Struggling	2-way ANOVA	Genotype, Condition	Genotype	(1, 35)	6.241	0.017	0.15	0.42
5H	Immobility	2-way ANOVA	Genotype, Condition	Genotype	(1, 35)	11.740	0.002	0.25	0.58
5I	Beclin1	3-way ANOVA	Genotype, Treatment, Condition	Genotype	(1, 72)	4.530	0.037	0.06	0.25
				Condition		6.160	0.015	0.08	0.29
				Geno x Treat		4.007	0.049	0.05	0.24
5J	Beclin1	2-way ANOVA	Genotype, Condition	Genotype	(1, 34)	6.407	0.016	0.16	0.43
5K	Atg12	3-way ANOVA	Genotype, Treatment, Condition	Genotype	(1, 72)	4.311	0.041	0.06	0.24
				Condition		5.487	0.022	0.07	0.28
				Geno x Treat		5.211	0.025	0.07	0.27

Figure	Measure	Test	Independent Factor(s)	Main Effect(s)	F df error	F-value	p-value	PES (partial Eta squared)	Estimated effect size: Cohen's f statistic
5L	Atg12	2-way ANOVA	Genotype, Condition	Genotype	(1, 34)	7.997	0.008	0.19	0.48
5M	pAkt(S473)/Akt	3-way ANOVA	Genotype, Treatment, Condition	Genotype	(1, 72)	6.023	0.017	0.08	0.29
				Treatment		7.465	0.008	0.09	0.32
				Geno x Treat		8.395	0.005	0.10	0.34
5N	pAkt(S473)/Akt (stress effect)	2-way ANOVA	Genoytpe, Treatment	Genotype	(1, 34)	4.184	0.049	0.11	0.35
				Treatment		4.711	0.037	0.12	0.37
5O	pAkt(S473)/Akt (treatment effect)	2-way ANOVA	Genoytpe, Condition	Genotype	(1, 34)	26.290	<0.0001	0.44	0.88
				Condition		6.962	0.012	0.17	0.45
5P	LC3B-II/I	3-way ANOVA	Genotype, Treatment,	Treatment	(1, 72)	4.873	0.030	0.06	0.26
				Geno x Cond		3.410	0.069	0.05	0.22
5Q	LC3B-II/I (stress effect)	2-way ANOVA	Genotype, Treatment	Genotype	(1, 34)	6.508	0.015	0.16	0.44
5R	LC3B-II/I (treatment effect)	2-way ANOVA	Genotype, Condition	Genotype	(1, 34)	3.251	0.080	0.09	0.31
S3B	LC3B-II actin	2-way ANOVA	Genotype, Treatment	Genotype	(1, 8)	9.043	0.017	0.53	1.06
				Treatment		19.429	0.002	0.71	1.56
S5C	pAkt (S473) / Akt	2-way ANOVA	Genotype, Treatment	Genotype	(1, 12)	17	0.001	0.59	1.20
				Treatment		5.767	0.018	0.49	0.98
S5E	pAkt (S473) / Akt	2-way ANOVA	Genotype, Treatment	Genotype	(1, 12)	17.787	<0.0001	0.75	1.72
S5F	LC3B-II / LC3B-I	2-way ANOVA	Genotype, Treatment	Genotype	(1, 12)	3.834	0.052	0.39	0.80
S6A	LC3B-II / LC3B-I	2-way ANOVA	Genotype, Treatment	Genotype	(1, 18)	14.75	<0.0001	0.62	1.28
				Treatment		3.243	0.063	0.27	0.60
S6C	puncta per cell	2-way ANOVA	Genotype, Treatment	Genotype	(1, 96)	107.763	<0.0001	0.53	1.06
				Treatment	(2, 96)	54.488	<0.0001	0.53	1.07
				Geno x Treat	(2, 96)	8.018	0.001	0.14	0.41

Figure	Measure	Test	Independent Factor(s)	Main Effect(s)	F df error	F-value	p-value	PES (partial Eta squared)	Estimated effect size: Cohen's f statistic
S6E	Beclin1	2-way ANOVA	Genotype, Treatment	Genotype	(1, 12)	9.547	0.009	0.44	0.89
	LC3B-II / LC3B-I	2-way ANOVA	Genotype, Treatment	Genotype	(1, 12)	95.477	<0.0001	0.89	2.82
				Treatment		182.467	<0.0001	0.97	5.50
				Geno x Treat		8.25	0.006	0.58	1.17
	Atg12	2-way ANOVA	Genotype, Treatment	Genotype	(1, 12)	205.655	<0.0001	0.95	4.15
				Treatment		6.636	0.011	0.53	1.05
S7	pAkt (S473) / Akt	1-way ANOVA	Treatment	Treatment	(3, 8)	7.482	0.01	0.74	1.67
	Beclin1	1-way ANOVA	Treatment	Treatment	(3, 8)	9.136	0.006	0.77	1.85
	Atg12	1-way ANOVA	Treatment	Treatment	(3, 8)	13.384	0.002	0.83	2.24
	LC3B-II / LC3B-I	1-way ANOVA	Treatment	Treatment	(3, 8)	18.228	0.001	0.87	2.61
S8A	LC3B-II / LC3B-I	2-way ANOVA	Treatment, BafA1	Treatment	(1, 8)	106.849	<0.0001	0.93	3.64
				BafA1		700.758	<0.0001	0.99	9.48
				Treat x BafA1		39.952	<0.0001	0.83	2.23
	LC3B-II / actin	2-way ANOVA	Treatment, BafA1	Treatment	(1, 8)	75.706	<0.0001	0.90	3.07
				BafA1		289.546	<0.0001	0.97	6.00
				Treat x BafA1		28.051	0.001	0.78	1.87
S8B	LC3B-II / LC3B-I	2-way ANOVA	Treatment, BafA1	Treatment	(1, 8)	40.289	<0.0001	0.83	2.24
				BafA1		230.714	<0.0001	0.97	5.33
				Treat x BafA1		8.989	0.017	0.53	1.06
	LC3B-II / actin	2-way ANOVA	Treatment, BafA1	Treatment	(1, 8)	508.928	<0.0001	0.99	8.10
				BafA1		698.884	<0.0001	0.99	9.48
				Treat x BafA1		289.945	<0.0001	0.97	6.00
S8C	LC3B-II / actin	2-way ANOVA	Treatment, BafA1	Treatment	(3, 36)	51.492	<0.0001	0.81	2.07
				BafA1	(1, 36)	33.454	<0.0001	0.48	0.96
				Treat x BafA1	(3, 36)	2.845	0.051	0.19	0.49
S9	Beclin1	2-way ANOVA	Genotype, Treatment	Genotype	(1, 8)	13.735	0.006	0.63	1.31
				Treatment		17.222	0.003	0.68	1.47

Figure	Measure	Test	Independent Factor(s)	Main Effect(s)	F df error	F-value	p-value	PES (partial Eta squared)	Estimated effect size: Cohen's f statistic
S10B	Beclin1 Ket	1-way ANOVA	Treatment	Treatment	(2, 6)	3.513	0.098	0.54	1.08
	LC3B-II / I Ket	1-way ANOVA	Treatment	Treatment	(2, 6)	4.245	0.071	0.59	1.19
	Beclin1 Sco	1-way ANOVA	Treatment	Treatment	(2, 6)	14.51	0.005	0.83	2.20
	LC3B-II / I Sco	1-way ANOVA	Treatment	Treatment	(2, 6)	2.781	0.14	0.48	0.96
S11A	corticosterone day22	2-way ANOVA	Genotype, Condition	Genotype	(1, 76)	6.46	0.013	0.08	0.29
				Condition		16.226	<0.0001	0.18	0.46
				Geno x Cond		4.519	0.037	0.06	0.24
S11B	Cort basal day43	3-way ANOVA	Genotype, Treatment, Condition	Genotype	(1, 68)	18.254	<0.0001	0.21	0.52
				Treatment		2.819	0.098	0.04	0.20
				Geno x Treat		2.990	0.088	0.04	0.21
S11C	corticosterone	2-way ANOVA	Genotype, Treatment	Genotype	(1, 32)	7.407	0.01	0.19	0.48
				Treatment		3.82	0.059	0.11	0.35
S11D	corticosterone	2-way ANOVA	Genotype, Condition	Genotype	(1, 33)	4.924	0.033	0.13	0.39
S11E	Cort response	3-way ANOVA	Genotype, Treatment, Condition	Genotype	(1, 71)	85.594	<0.0001	0.55	1.10
S11F	Cort recovery	3-way ANOVA	Genotype, Treatment,	Genotype	(1, 73)	67.998	<0.0001	0.48	0.96
				Condition		10.703	0.002	0.13	0.38
S12F	Beclin1	2-way ANOVA	Genotype, Treatment	Genotype	(1, 8)	11.957	0.009	0.60	1.22
				Geno x Treat		15.314	0.004	0.66	1.38
	Vps34	2-way ANOVA	Genotype, Treatment	Genotype	(1, 8)	52.354	<0.0001	0.87	2.55
				Treatment		27.295	0.001	0.77	1.85
				Geno x Treat		15.794	0.004	0.66	1.41
	pAkt (S473) / Akt DEX	2-way ANOVA	Genotype, Treatment	Genotype	(1, 8)	10.232	0.012	0.56	1.14
				Treatment		15.608	0.004	0.66	1.40
