# Supplementary material for: Association of FKBP51 with Priming of Autophagy Pathways and Mediation of Antidepressant Treatment Response: Evidence in Cells, Mice, and Humans
Source: PLoS Med. 2014 Nov 11;11(11):e1001755. doi: 10.1371/journal.pmed.1001755 (PMC4227651; doi:10.1371/journal.pmed.1001755)
Supplement: Table S2 — Details of the results of the regression analyses. (PDF) [file pmed.1001755.s014.pdf]

Table S2, Gassen et al.

Figure	y-axis	x-axis	r-value	p-value
6A	Beclin1 expr.	FKBP51 expr.	0.726	0.00029
6B	pAkt/Akt expr.	FKBP51 expr.	-0.537	0.015
6C	LC3B-II/I expr.	FKBP51 expr.	0.423	0.056
6D	Atg12 expr.	FKBP51 expr.	0.452	0.046
7A	Beclin1 change [%]	FKBP51 Change [%]	0.737	0.002
7B	pAkt/Akt change [%]	FKBP51 Change [%]	-0.497	0.07
7C	Beclin1 change [%]	FKBP51 expr.	0.703	0.002
7D	pAkt/Akt change [%]	FKBP51 expr.	-0.467	0.068
7E	LC3B-II/I change [%]	FKBP51 expr.	0.626	0.009
7F	Atg12 change [%]	FKBP51 expr.	0.703	0.002
8A	Beclin1 expr.	clinical AD response [% change]	0.521	0.002
8B	pAkt/Akt expr.	clinical AD response [% change]	-0.515	0.003
8C	FKBP51 expr.	clinical AD response [% change]	0.631	<0.0001
8D	Beclin1 expr.	FKBP51 expr.	0.415	0.018
8E	pAkt/Akt expr.	FKBP51 expr.	-0.377	0.033
8F	LC3B-II/I expr.	FKBP51 expr.	0.39	0.027
9A	cellular AD response Beclin1 [% change]	clinical AD response [% change]	0.572	0.003
9B	cellular AD response Beclin1 [% change]	clinical AD response [% change]	0.569	0.004
9C	cellular AD response Beclin1 [% change]	clinical AD response [% change]	0.454	0.026
9D	cellular AD response pAkt/Akt [% change]	clinical AD response [% change]	-0.416	0.006

Figure	y-axis	x-axis	r-value	p-value
9E	cellular AD response pAkt/Akt [% change]	clinical AD response [% change]	-0.355	0.021
9F	cellular AD response pAkt/Akt [% change]	clinical AD response [% change]	0.052	0.746
9G	cellular AD response LC3BII/I [% change]	clinical AD response [% change]	0.374	0.06
9H	cellular AD response LC3BII/I [% change]	clinical AD response [% change]	0.453	0.02
9I	cellular AD response LC3BII/I [% change]	clinical AD response [% change]	0.189	0.355
S12H	Beclin1 change [%] (AMI)	FKBP51 expr.	0.594	0.015
S12H	pAkt/Akt change [%] (AMI)	FKBP51 expr.	-0.523	0.038
S12H	LC3B-II/I change [%] (AMI)	FKBP51 expr.	0.727	0.001
S12H	Atg12 change [%] (AMI)	FKBP51 expr.	0.234	0.383
S12H	Beclin1 change [%] (FLX)	FKBP51 expr.	0.336	0.204
S12H	pAkt/Akt change [%] (FLX)	FKBP51 expr.	-0.529	0.035
S12H	LC3B-II/I change [%] (FLX)	FKBP51 expr.	0.782	<0.0001
S12H	Atg12 change [%] (FLX)	FKBP51 expr.	0.26	0.331
