# Supplementary material for: Association of FKBP51 with Priming of Autophagy Pathways and Mediation of Antidepressant Treatment Response: Evidence in Cells, Mice, and Humans
Source: PLoS Med. 2014 Nov 11;11(11):e1001755. doi: 10.1371/journal.pmed.1001755 (PMC4227651; doi:10.1371/journal.pmed.1001755)
Supplement: Text S1 — STROBE statement. (DOCX) [file pmed.1001755.s015.docx]

The ARRIVE Guidelines Checklist

Animal Research: Reporting In Vivo Experiments

EM

|  | ITEM | RECOMMENDATION | SECTION/PARAGRAPH |
| --- | --- | --- | --- |
| Title | 1 | Provide as accurate and concise a description of the content of the article as possible | Association of FKBP51 with Priming Autophagy Pathways and Mediating  Antidepressant Treatment Response: Evidence in Cells, Mice and Humans |
| Abstract | 2 | Provide an accurate summary of the background, research objectives, including details of the species or strain of animal used, key methods, principal findings and conclusions of the study. | Abstract, pages 2-3 |
| Introduction |  |  |  |
| Background | 3 | a. Include sufficient scientific background (including relevant references to previous  work) to understand the motivation and context for the study, and explain the  experimental approach and rationale.  b. Explain how and why the animal species and model being used can address the  scientific objectives and, where appropriate, the study’s relevance to human biology. | Introduction, pages 4-6 |
| Objectives | 4 | Clearly describe the primary and any secondary objectives of the study, or specific  hypotheses being tested. | Introduction, page 5, last paragraph – page 6 |
| Methods |  |  |  |
| Ethical statement | 5 | Indicate the nature of the ethical review permissions, relevant licences (e.g. Animal  [Scientific Procedures] Act 1986), and national or institutional guidelines for the care and  use of animals, that cover the research. | Page 17 |
| Study design | 6 | For each experiment, give brief details of the study design including:  a. The number of experimental and control groups.  b. Any steps taken to minimise the effects of subjective bias when allocating animals to  treatment (e.g. randomisation procedure) and when assessing results (e.g. if done,  describe who was blinded and when).  c. The experimental unit (e.g. a single animal, group or cage of animals).  A time-line diagram or flow chart can be useful to illustrate how complex study designs  were carried out. | In detail described in pages 10 – 15, Fig. 4A |
| Experimental procedures | 7 | For each experiment and each experimental group, including controls, provide precise  details of all procedures carried out. For example:  a. How (e.g. drug formulation and dose, site and route of administration, anaesthesia  and analgesia used [including monitoring], surgical procedure, method of euthanasia).  Provide details of any specialist equipment used, including supplier(s).  b. When (e.g. time of day).  c. Where (e.g. home cage, laboratory, water maze).  d. Why (e.g. rationale for choice of specific anaesthetic, route of administration, drug  dose used). | In detail described in pages 10 – 15 |
| Experimental animals | 8 | a. Provide details of the animals used, including species, strain, sex, developmental  stage (e.g. mean or median age plus age range) and weight (e.g. mean or median  weight plus weight range).  b. Provide further relevant information such as the source of animals, international strain  nomenclature, genetic modification status (e.g. knock-out or transgenic), genotype,  health/immune status, drug or test naïve, previous procedures, etc. | In detail described in pages 10 – 15 |
| Housing and husbandry | 9 | Provide details of:  a. Housing (type of facility e.g. specific pathogen free [SPF]; type of cage or housing;  bedding material; number of cage companions; tank shape and material etc. for fish).  b. Husbandry conditions (e.g. breeding programme, light/dark cycle, temperature,  quality of water etc for fish, type of food, access to food and water, environmental  enrichment).  c. Welfare-related assessments and interventions that were carried out prior to, during,  or after the experiment. | In detail described in pages 10 – 15 |
| Sample size | 10 | a. Specify the total number of animals used in each experiment, and the number of  animals in each experimental group.  b. Explain how the number of animals was arrived at. Provide details of any sample size  calculation used.  c. Indicate the number of independent replications of each experiment, if  relevant. | a. Legend figure 1 and 5  b. The number of animals used was based on previous experiments and sample size estimations based on expected effect size  c. not applicable |
| Allocating animals to experimental groups | 11 | a. Give full details of how animals were allocated to experimental groups, including  randomisation or matching if done.  b. Describe the order in which the animals in the different experimental groups were  treated and assessed. | a. Animals were assigned to the experimental groups in a semi-randomized manner  b. the order of treatment and assessment of behavioral outcomes was semi-randomized |
| Experimental outcomes | 12 | Clearly define the primary and secondary experimental outcomes assessed (e.g. cell  death, molecular markers, behavioural changes). | pages 10 – 15 |
| Statistical methods | 13 | a. Provide details of the statistical methods used for each analysis.  b. Specify the unit of analysis for each dataset (e.g. single animal, group of animals,  single neuron).  c. Describe any methods used to assess whether the data met the assumptions of the  statistical approach. | Statistics section page 17-18 |
| Results |  |  |  |
| Baseline data | 14 | For each experimental group, report relevant characteristics and health status of animals  (e.g. weight, microbiological status, and drug or test naïve) prior to treatment or testing.  (This information can often be tabulated). | All experimental animals are drug and test naïve before the start of the experiment. |
| Numbers analysed | 15 | a. Report the number of animals in each group included in each analysis. Report absolute  numbers (e.g. 10/20, not 50%†).  b. If any animals or data were not included in the analysis, explain why. | a. Legend figure 1 and 5  b. page 14 first paragraph |
| Outcomes and estimation | 16 | Report the results for each analysis carried out, with a measure of precision (e.g.  standard error or confidence interval). | Results are presented as box plots or correlation plots. Detailed information of precision and effect size is provided as elaborated in several rounds of revision. |
| Adverse events | 17 | a. Give details of all important adverse events in each experimental group.  b. Describe any modifications to the experimental protocols made to reduce adverse  events. | Not applicable. No adverse events occurred during the experiments. |
| Discussion |  |  |  |
| Interpretation / scientific implications | 18 | a. Interpret the results, taking into account the study objectives and hypotheses, current  theory and other relevant studies in the literature.  b. Comment on the study limitations including any potential sources of bias, any  limitations of the animal model, and the imprecision associated with the results†.  c. Describe any implications of your experimental methods or findings for the  replacement, refinement or reduction (the 3Rs) of the use of animals in research. | a. Discussion, page 30 – 32  b. Discussion, page 32 upper half  c. not applicable |
| Generalisability / translation | 19 | Comment on whether, and how, the findings of this study are likely to translate to other  species or systems, including any relevance to human biology. | Our study is a very clear example of high translatability, as we extend our findings directly to humans. This is discussed: page 30-33 |
| Funding | 20 | List all funding sources (including grant number) and the role of the funder(s) in the  study. | Page 41 |

ECOMMENDATION

Section/

Paragraph

INTRODUCTION
