# Supplementary material for: Association of FKBP51 with Priming of Autophagy Pathways and Mediation of Antidepressant Treatment Response: Evidence in Cells, Mice, and Humans
Source: PLoS Med. 2014 Nov 11;11(11):e1001755. doi: 10.1371/journal.pmed.1001755 (PMC4227651; doi:10.1371/journal.pmed.1001755)
Supplement: Text S3 — Extended information on Western blot quantification. (DOCX) [file pmed.1001755.s017.docx]

Extended information on Western blot quantification

To Gassen et al. Association of FKBP51 with Priming Autophagy Pathways and Mediating

Antidepressant Treatment Response: Evidence in Cells, Mice and Humans

Most of the Western blot examples in the figures display several proteins that are derived from separate blots. Actin was routinely detected (fluorescence-coupled antibody) at the same time as the protein of interest (different size, ECL) and used for normalization. Only one Actin example is provided in the figures.

Some figures show blots where sequential detection has been applied. Figure S2 provides an example of the different procedures. In panel A, Atg12 and pAkt^S473^ are in separate boxes, but they are in the same box in panel B. The blots were routinely probed with pAkt^S473^ antibody first (and the picture was recorded) and the same blots were then probed with Atg12 antibody (without stripping). If the two bands were clearly distinguishable, this blot was used for quantification of both pAkt^S473^ and Atg12 (panels in B). If the bands appeared too close, the first recording for pAkt^S473^ was used, and a separate gel was run for blotting and detecting Atg12 (panels in A).

In some cases, sequential detection on the same blot was possible for proteins that run very close on the gel if the different protein abundance/antibody sensitivity results in very different times needed to record the signal. For example, Beclin1 and Akt are in many cases on the same blot, detected sequentially. Detection of Beclin1 required recording times of about 3 minutes in the Imaging system, while detection of Akt took about 2 seconds. Thus, the reasoning was that even if some (or even all) signal of Beclin1 should have remained after washing and subsequent probing for Akt, the contribution of the signal of Beclin1 after 2 seconds is negligible (certainly below variations inherent in the overall Western procedure).
